# Intraflagellar transport-associated CCDC92 is required for spermiogenesis and male fertility in mice

**DOI:** 10.1093/jmcb/mjaf022

**Published:** 2025-08-05

**Authors:** Yue Lu, Xirui Zi, Qian Lyu, Qingchao Li, Hanxiao Yin, Yinghao Wang, Qijun Chen, Bingkun Kang, Shanshan Nai, Jun Zhou, Huijie Zhao, Ting Song

**Affiliations:** Center for Cell Structure and Function, Shandong Provincial Key Laboratory of Animal Resistance Biology, College of Life Sciences, Shandong Normal University, Jinan 250014, China; Center for Cell Structure and Function, Shandong Provincial Key Laboratory of Animal Resistance Biology, College of Life Sciences, Shandong Normal University, Jinan 250014, China; Center for Cell Structure and Function, Shandong Provincial Key Laboratory of Animal Resistance Biology, College of Life Sciences, Shandong Normal University, Jinan 250014, China; Center for Cell Structure and Function, Shandong Provincial Key Laboratory of Animal Resistance Biology, College of Life Sciences, Shandong Normal University, Jinan 250014, China; State Key Laboratory of Medicinal Chemical Biology, Haihe Laboratory of Cell Ecosystem, College of Life Sciences, Nankai University, Tianjin 300071, China; Center for Cell Structure and Function, Shandong Provincial Key Laboratory of Animal Resistance Biology, College of Life Sciences, Shandong Normal University, Jinan 250014, China; Center for Cell Structure and Function, Shandong Provincial Key Laboratory of Animal Resistance Biology, College of Life Sciences, Shandong Normal University, Jinan 250014, China; Center for Cell Structure and Function, Shandong Provincial Key Laboratory of Animal Resistance Biology, College of Life Sciences, Shandong Normal University, Jinan 250014, China; Center for Cell Structure and Function, Shandong Provincial Key Laboratory of Animal Resistance Biology, College of Life Sciences, Shandong Normal University, Jinan 250014, China; Center for Cell Structure and Function, Shandong Provincial Key Laboratory of Animal Resistance Biology, College of Life Sciences, Shandong Normal University, Jinan 250014, China; State Key Laboratory of Medicinal Chemical Biology, Haihe Laboratory of Cell Ecosystem, College of Life Sciences, Nankai University, Tianjin 300071, China; Center for Cell Structure and Function, Shandong Provincial Key Laboratory of Animal Resistance Biology, College of Life Sciences, Shandong Normal University, Jinan 250014, China; Center for Cell Structure and Function, Shandong Provincial Key Laboratory of Animal Resistance Biology, College of Life Sciences, Shandong Normal University, Jinan 250014, China

**Keywords:** CCDC92, male infertility, spermiogenesis, manchette, intraflagellar transport, microtubule inner proteins

## Abstract

The differentiation of a round spermatid into a streamlined sperm cell involves a series of remarkable morphological changes, such as sperm head shaping and flagellum formation. However, the underlying mechanism of spermatid shaping remains unclear. In this study, we find that CCDC92 deficiency in mice leads to severe abnormalities of the sperm head and flagellum and causes male infertility. Ultrastructural analyses of testicular elongating *Ccdc92* knockout spermatids reveal severely deformed manchette structures. The manchette defects impair the subsequent sperm nucleus elongation and acrosome anchoring, resulting in misshapen rod-like nuclei and detached acrosomes. Molecularly, CCDC92 interacts with intraflagellar transport (IFT) complex components and colocalizes with IFT proteins at the manchette in developing spermatids. Quantitative proteomics further reveals the requirement of CCDC92 for proper flagellar distribution of axonemal microtubule inner proteins. Our findings demonstrate an essential role of CCDC92 in regulating spermatid shaping and provide novel insights into the pathology of male infertility.

## Introduction

Male infertility is a worldwide health issue affecting >30 million men globally, a situation that commonly results from the decrease or lack of functional spermatozoa (sperm) in the semen (Agarwal et al., 2015; Huang et al., 2023). Spermatozoa are highly differentiated cells with particular features related to their function: a head to carry and protect the genome and a tail or flagellum to power sperm motility. The sperm head mainly consists of a limited amount of cytoplasm, highly condensed chromatin, and a unique membranous acrosome. The acrosome is located at the anterior region of the head and contains specific hydrolytic enzymes that digest the zona pellucida of the oocyte for sperm penetration. Failure of acrosome formation affects the penetration process and leads to male infertility. Additionally, many cases of male infertility are associated with aberrant sperm flagella, including absent, short, coiled, and irregular flagella, collectively referred to as multiple morphological abnormalities of the sperm flagella (MMAF) (Ben Khelifa et al., 2014). Growing evidence indicates that gene mutations, including single- and multiple-gene mutations, can impair sperm development (Lehti and Sironen, 2017).

Spermatogenesis is the cellular process that produces mature functional spermatozoa. Spermatogenesis occurs within the seminiferous tubules of the testis. It can be divided into three phases: the mitotic division of spermatogonia, meiosis of spermatocytes, and post-meiotic differentiation of spermatids (also called spermiogenesis) (Ramm and Scharer, 2014; Griswold, 2016; Neto et al., 2016). Throughout spermatogenesis, remarkable morphological changes are committed in spermiogenesis, including chromatin condensation, acrosome formation, cytoplasm reduction, mitochondria relocalization, and flagellum development (O'Donnell, 2014). The manchette, a microtubule-based structure, is transiently assembled during sperm head shaping and undergoes rapid disassembly during spermiogenesis (O'Donnell, 2014; Lehti and Sironen, 2016). The manchette microtubule mantle is embedded in the perinuclear ring and extends toward the caudal side. In addition, actin filaments are also associated with the manchette (Sun et al., 2011). Consistent with the concurrent appearance of the manchette with nuclear shaping, the manchette plays an essential role in nuclear shaping and flagellum formation during spermiogenesis (Lehti and Sironen, 2016; Hu et al., 2023).

The sperm flagellum is a modified motile cilium, which exhibits a conserved axoneme arrangement with nine microtubule doublets (MTDs) surrounding a central microtubule pair (9+2 structure) but possesses specific accessory structures, including the mitochondrial sheath (MS), the fibrous sheath (FS), and ODFs (Linck et al., 2016; Lyu et al., 2024). Based on the distribution of accessory structures, a sperm flagellum can be divided into three major parts: the midpiece, the principal piece, and the endpiece. The midpiece is next to the sperm head, with its axoneme surrounded by the MS and nine ODFs. The principal piece is the most extended portion of the sperm tail, characterized by the presence of two longitudinal columns of the FS and seven ODFs. The endpiece is adjacent to the principal piece and contains only the axoneme surrounded by the plasma membrane (Lehti and Sironen, 2017; Pereira and Sousa, 2023). As the core structure, the axonemal microtubules are decorated with multiple microtubule inner proteins (MIPs) on the luminal side for axoneme stability and motility (Gui et al., 2021; Leung et al., 2023; Tai et al., 2023; Zhou et al., 2023). However, the assembly mechanisms of MIPs are still unclear.

The development of sperm flagella relies on the intraflagellar transport (IFT), a bidirectional movement of multiprotein complexes essential for cilia and flagella. The IFT system consists of two large protein complexes, the IFT-A and IFT-B complexes. The IFT-B complex is responsible for the anterograde transport that carries cargoes to the tip of cilia and flagella, whereas the IFT-A complex returns IFT and ciliary proteins to the cell body (retrograde transport) (Ishikawa and Marshall, 2017; Klena and Pigino, 2022). During spermiogenesis, another distinct transport system exists, the intramanchette transport, which utilizes the transient manchette to transport accessory structural proteins to the base of the developing flagellum, where those proteins are further delivered into the flagellum through IFT (Kierszenbaum, 2002; Lehti and Sironen, 2016). Both transport systems share multiple components, such as IFT complex components and motor proteins. Several IFT components, including IFT20, IFT88, IFT140, and IFT172, have been shown to localize to the manchette (Zhang et al., 2018, 2020; Yap et al., 2022). Disruption of IFT complexes in male germ cells affects spermiogenesis, resulting in male infertility in mice (Zhang, 2021).

The mouse *Ccdc92* gene is located on chromosome 5, whereas the human orthologue *CCDC92* is located on chromosome 12q24. Multiple genome-wide association studies have linked this genomic region to the pathogenesis of human insulin resistance and coronary heart disease (Klarin et al., 2017; Lotta et al., 2017; Zhao et al., 2017). Recent phenotypic analysis of *Ccdc92* knockout (KO) mice has demonstrated that CCDC92 is involved in obesity and insulin resistance by mediating the inflammatory response in white adipose tissue (Ren et al., 2023). In addition, CCDC92 can regulate lipid homeostasis to promote podocyte lipo-toxicity, contributing to diabetic kidney disease (Zuo et al., 2024). As a coiled-coil-containing protein, CCDC92 interacts with CEP164 at the centriolar distal end and can be phosphorylated by TTBK2 (Chaki et al., 2012; Bernatik et al., 2020), both proteins playing essential roles in ciliogenesis (Graser et al., 2007; Goetz et al., 2012). However, it remains unclear whether CCDC92 is associated with ciliogenesis.

Here, we report that CCDC92 is essential for sperm head shaping and flagellum formation during spermiogenesis. Despite no apparent morphological alterations in *Ccdc92* KO mice, CCDC92 deficiency leads to malformed sperm heads and defective flagella in mature spermatozoa, resulting in male infertility. Through mass spectrometry-based analysis, we identify IFT-B proteins as CCDC92 interactors and show that CCDC92 can traffic with IFT along the axoneme. CCDC92 is located on the manchette during spermatid development and regulates the manchette for sperm head shaping. Quantitative proteomics further reveals the requirement of CCDC92 for the flagellar distribution of axonemal MIPs. Overall, our investigations elucidate *Ccdc92* as a causative gene of male infertility in mice and establish functional links between CCDC92, IFT, and axonemal MIPs during spermiogenesis.

## Results

### CCDC92 deficiency leads to male infertility in mice

**Figure 1:**
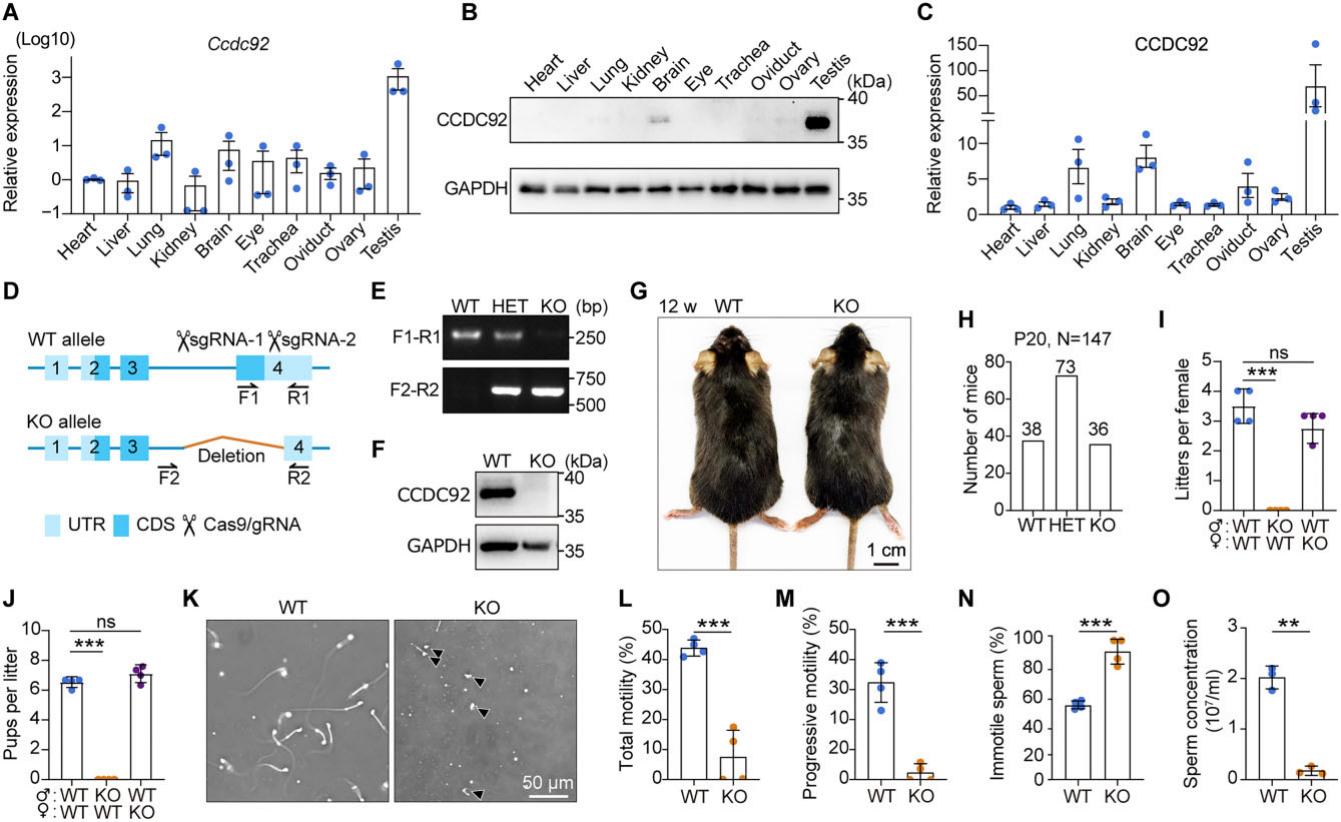
*Ccdc92* KO mice are male infertile. (**A**) Real-time PCR analysis showing the relative expression of *Ccdc92* in various mouse tissues, normalized using the corresponding *Gapdh* as the reference gene and baseline 1 (heart) as the reference sample (△△CT method). Data are from three independent biological repeats and presented on a logarithmic scale (log_10_) as mean ± standard error of the mean. (**B** and **C**) Immunoblotting and quantification showing the expression levels of CCDC92 in various mouse tissues. The CCDC92 band intensity was normalized to the corresponding GAPDH using ImageJ. (**D**) A schematic diagram of WT and *Ccdc92* KO alleles. The genomic positions of primers (F1, R1, F2, and R2) used for genotyping are indicated. UTR, untranslated region; CDS, coding sequence. (**E**) Genotyping of WT, heterozygous (HET), and *Ccdc92* KO mice. (**F**) Immunoblotting showing the depletion efficiency in the *Ccdc92* KO testes. GAPDH was used as a loading control. (**G**) Representative images of WT and *Ccdc92* KO mice at 12 weeks of age. (**H**) Number of mice of the indicated genotypes at postnatal day 20 (P20). (**I** and **J**) Fertility assay of WT and *Ccdc92* KO mice (*n* = 4 mice per genotype). (**K**–**N**) CASA of epididymis sperm isolated from WT and *Ccdc92* KO mice (*n* = 4 mice per genotype). (**O**) Epididymal sperm counts using a hemocytometer (*n* = 3 mice per genotype). Data in **C, I, J**, and **L**–**O** are presented as mean ± SD. Unpaired two-tailed *t*-test was performed. ns, not significant; **P < 0.01; ***P < 0.001.

To create a comprehensive overview of CCDC92 expression, we examined the tissue distribution pattern of CCDC92 in mice. A panel of tissues was collected from 8-week-old wild-type (WT) mice and subjected to real-time polymerase chain reaction (PCR) and immunoblotting. As shown in Figure 1A, *Ccdc92* mRNA expression was the highest in the testis, and lower expression levels were present in the lung and brain. Consistently, immunoblotting revealed the highest expression level of CCDC92 in the testis (Figure 1B and C). This expression pattern suggests that CCDC92 may play a role in the male reproductive system.

To define the function of CCDC92 *in vivo*, a *Ccdc92* KO mouse model was generated using CRISPR–Cas9 with dual single-guide RNAs (sgRNAs) (Figure 1D). Genome deletions introduced with sgRNAs were identified by PCR (Figure 1E). Immunoblotting further confirmed a complete loss of CCDC92 in the *Ccdc92* KO testis tissue (Figure 1F). *Ccdc92* KO mice were born at Mendelian ratios with no gross morphological abnormalities (Figure 1G and H). The genotypes were indistinguishable with respect to behavior in standard cage environments. Given the highest expression of CCDC92 in the testis, we sought to test the fertility of *Ccdc92* KO males. *Ccdc92* KO and WT males or females were separately mated to C57BL/6J mice. Strikingly, matings between *Ccdc92* KO males and WT females within the breeding period (3 months) did not produce any offspring. By contrast, matings involving WT males with WT females resulted in comparable numbers of offspring (Figure 1I and J). We also noted that *Ccdc92* KO females were fertile, based on the progeny produced from *Ccdc92* KO females bred to WT males (Figure 1I and J).

To understand the underlying fertility defect in *Ccdc92* KO males, computer-assisted sperm analysis (CASA) was conducted to evaluate sperm motility extracted from the cauda epididymis of adult WT and *Ccdc92* KO mice. Representative images and videos from CASA illustrated the stark difference in motility and morphology between WT and *Ccdc92* KO spermatozoa (Figure 1K; Supplementary Video S1). The percentage of total motile *Ccdc92* KO spermatozoa was significantly reduced compared with WT spermatozoa (Figure 1L). Sperm cells with progressive motility were consistently decreased in *Ccdc92* KO mice, and the percentage of immotile spermatozoa was significantly increased (Figure 1M and N). In addition, the counts of spermatozoa obtained from *Ccdc92* KO cauda epididymis were drastically reduced (Figure 1O). CASA also revealed that a significant proportion of the *Ccdc92* KO spermatozoa displayed abnormal morphology (Figure 1K; Supplementary Video S1), indicative of teratozoospermia in *Ccdc92* KO males. These data demonstrate that CCDC92 is exclusively essential for sperm development and male fertility in mice.

### Loss of CCDC92 causes epididymal sperm abnormalities

**Figure 2:**
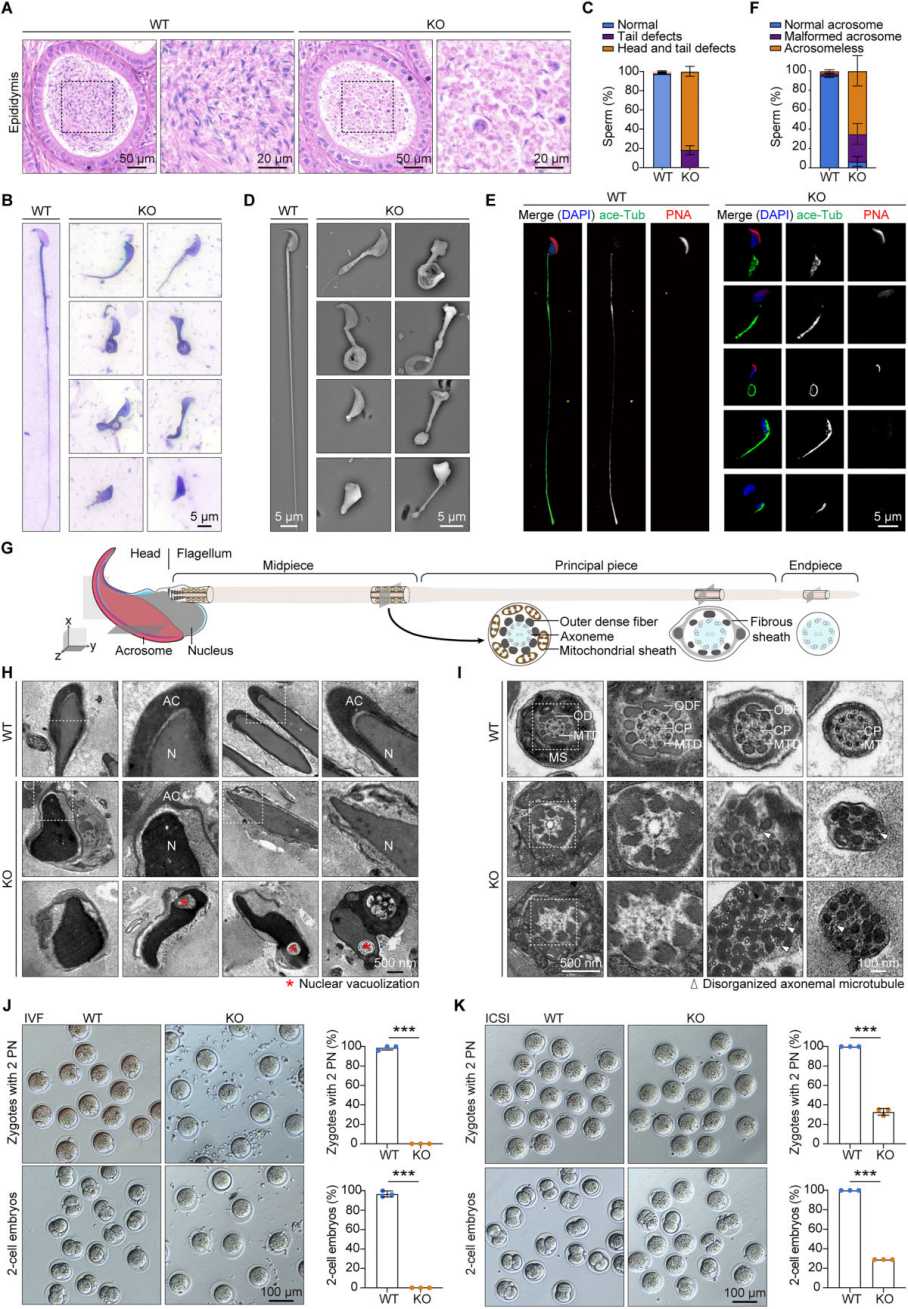
CCDC92 deficiency leads to sperm abnormalities. (**A**) HE staining of WT and *Ccdc92* KO epididymis sections. Magnified images of the dashed boxed regions are shown on the right. (**B**–**F**) Giemsa (**B** and **C**, *n* = 3 mice per genotype), scanning electron microscopy (**D**), and immunofluorescence (**E** and **F**, *n* = 3 mice per genotype) analyses of sperm cells released from WT and *Ccdc92* KO epididymis. For immunofluorescence analysis, sections were stained with an acetylated α-tubulin (ace-Tub) antibody, DAPI, and Alexa Fluor 568-conjugated PNA. (**G**) Structural features of the mouse sperm. The sperm consists of two main parts: the head and the flagellum. The acrosome covers the anterior portion of the nucleus. The sperm flagellum is divided into three regions: the midpiece, the principal piece, and the endpiece. The flagellar axoneme displays a ‘9+2’ microtubule arrangement. Key structural components of each region are highlighted: the axoneme, ODF, MS, and FS. (**H** and **I**) TEM images of the heads (**H**) and flagella (**I**) of the spermatozoa released from WT and *Ccdc92* KO epididymis. Magnified images of the dashed boxed regions are shown on the right. Note the detached acrosome (AC), the abnormal nuclear (N) vacuolization, and the disorganized ODFs and axonemal MTDs with central pair (CP) microtubules in *Ccdc92* KO sperm cells. (**J** and **K**) Representative images and quantifications of zygotes with 2 PN and embryos at the 2-cell stage from IVF (**J**) and ICSI (**K**) (*n* = 3 mice per genotype). Data are presented as mean ± SD. Unpaired two-tailed *t*-test was performed. ***P < 0.001.

To further assess the sperm defects revealed by CASA, multiple techniques were utilized to examine the epididymides and spermatozoa of *Ccdc92* KO mice. Hematoxylin–eosin (HE) staining of WT and *Ccdc92* KO epididymis sections showed that the WT cauda epididymis was filled with sperm cells. In contrast, the *Ccdc92* KO cauda epididymis contained significantly fewer sperm cells (Figure 2A). Trypan blue–Giemsa staining was thus performed to evaluate the sperm morphology. All the *Ccdc92* KO spermatozoa examined displayed typical MMAF, including absent, coiled, and short flagella, whereas only 2.3% of WT spermatozoa had defective flagella (Figure 2B and C). Scanning electron microscopy confirmed morphological abnormalities in *Ccdc92* KO spermatozoa (Figure 2D).

In addition to deformed flagella, 82.3% of the *Ccdc92* KO spermatozoa exhibited abnormal morphology in the sperm head, such as small, round, or irregular heads (Figure 2B–D). To better understand this phenomenon, peanut agglutinin (PNA) staining was used to assess the sperm acrosome. PNA is a plant lectin that specifically stains the outer membrane and the contents of acrosomes at low concentrations (Nakata et al., 2017). Most spermatozoa obtained from WT cauda epididymis showed a maximum PNA fluorescence in the apical ridge area of the sperm head (Figure 2E and F). By contrast, the distribution of PNA signals was nonuniformly altered in the *Ccdc92* KO spermatozoa (Figure 2E). In 28.3% of the *Ccdc92* KO spermatozoa, the acrosomal morphology changed from the sickle shape to the irregular cap-like form (Figure 2E and F). In addition, 65% of the *Ccdc92* KO spermatozoa showed no PNA staining, and small or irregularly elongated heads were frequently observed (Figure 2E and F), indicative of impaired head shaping in *Ccdc92* KO spermatozoa.

To precisely assess the sperm ultrastructure (Figure 2G), transmission electron microscopy (TEM) analysis of the cauda epididymis was conducted to examine the ultrastructural differences between WT and *Ccdc92* KO spermatozoa. In WT epididymal spermatozoa, the acrosome full of a homogenous acrosomal matrix was closely adjacent to the plasma membrane covering the anterior portion of the sperm head (Figure 2H). In stark contrast to the highly ordered acrosome arrays in WT spermatozoa, the majority of *Ccdc92* KO spermatozoa with acrosomes had an enlarged subacrosomal space, which was often combined with the presence of nuclear vacuolization (Figure 2H). Spermatozoa with an acrosome lacking contents or with no acrosome were also observed in the *Ccdc92* KO epididymis (Figure 2H). As for the flagellar ultrastructure, the *Ccdc92* KO sperm flagella displayed a loss of ‘9+2’ microtubule arrangement with ODFs translocated or overnumbered (>9 ODFs) (Figure 2I). These data reveal that loss of CCDC92 in mice leads to sperm ultrastructural abnormalities in heads and flagella.

We next examined whether the *Ccdc92* KO spermatozoa could fertilize mature eggs. The *Ccdc92* KO sperm were released from the epididymis, and those with overall normal head appearance were selected for *in vitro* fertilization (IVF) and intracytoplasmic sperm injection (ICSI). Fertilization was assessed by the presence of two pronuclei (2 PN) and the percentage of two-cell embryos (2-cell stage). After mixing the eggs with the WT sperm via the IVF procedure, most eggs could divide and develop into the 2-cell stage. In stark contrast, no fertilization occurred in the *Ccdc92* KO sperm samples (Figure 2J). Interestingly, the ICSI procedure, in which a sperm cell is directly injected into the egg cytoplasm, partially improved the fertilization ratio in *Ccdc92* KO samples (Figure 2K). These results suggest that ICSI is effective to a certain extent in rescuing *Ccdc92*-associated male infertility.

### CCDC92 is required for testicular spermiogenesis

**Figure 3:**
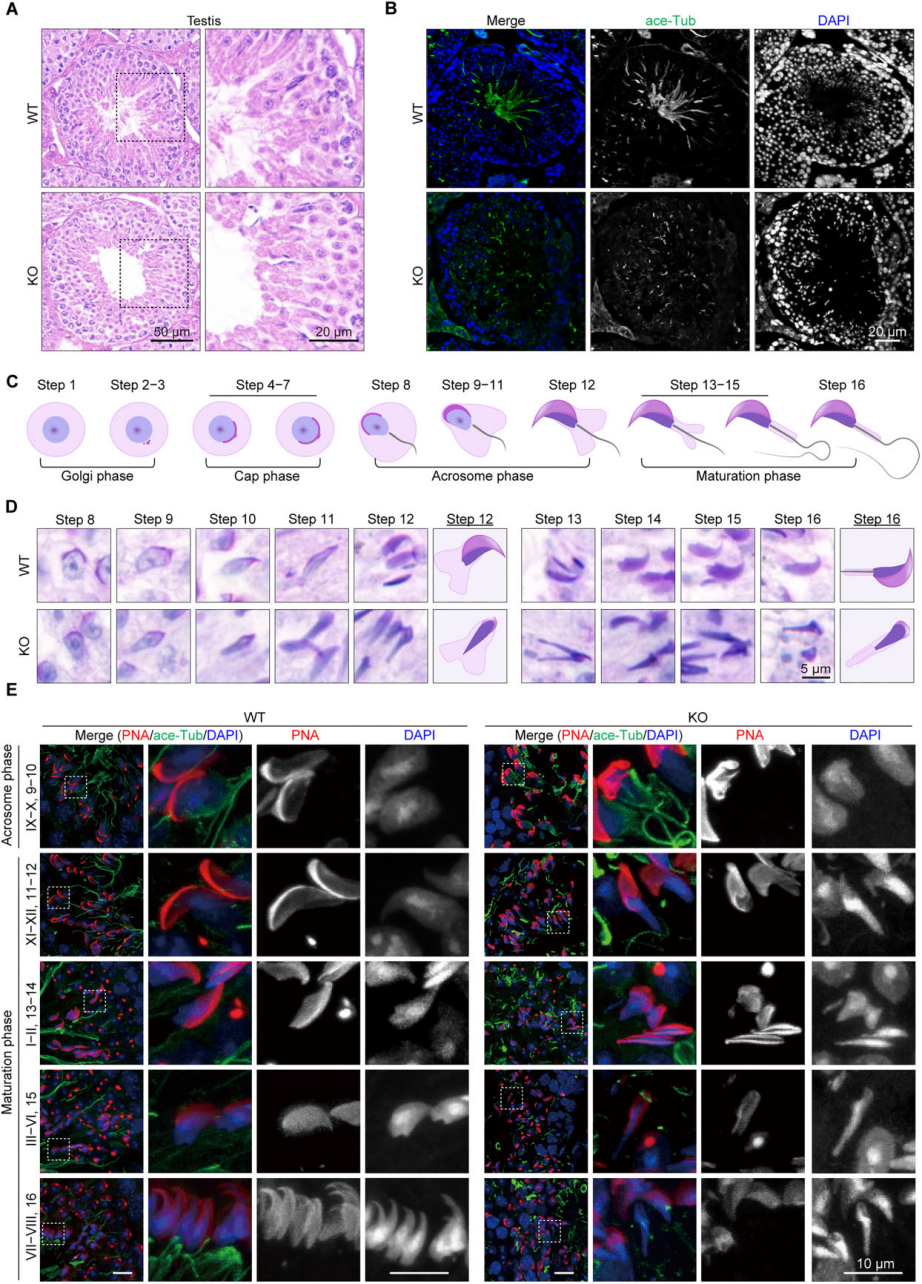
CCDC92 is essential for proper spermiogenesis in seminiferous tubules. (**A**) HE staining of WT and *Ccdc92* KO testis sections. Magnified images of the dashed boxed regions are shown on the right. (**B**) Immunofluorescence staining of WT and *Ccdc92* KO testis sections with the ace-Tub antibody and DAPI. (**C**) A diagram of spermiogenesis. Round spermatids undergo four different phases (Golgi, cap, acrosome, and maturation phases) to differentiate into mature spermatozoa. (**D**) PAS and hematoxylin staining of WT and *Ccdc92* KO testis sections revealing significant defects in the later steps (steps 10–16) of spermiogenesis in *Ccdc92* KO spermatids. (**E**) Fluorescence images of seminiferous tubules containing spermatids at the acrosome and maturation phases in WT and *Ccdc92* KO testis sections. Sections were stained with the ace-Tub antibody, Alexa Fluor 568-conjugated PNA, and DAPI. Magnified images of the dashed boxed regions are shown on the right.

Given the severe morphological defects observed in *Ccdc92* KO spermatozoa, we set out to determine CCDC92 expression in the testes at different developmental stages. Real-time PCR and immunoblotting showed that CCDC92 was highly expressed in the developing spermatids (Supplementary Figure S1A and B). Next, we examined the WT and *Ccdc92* KO testes to determine the onset of spermatogenic defects. Upon dissection, no apparent morphological abnormalities in *Ccdc92* KO testes were observed (Supplementary Figure S1C). The testis mass relative to total body weight was comparable between WT and *Ccdc92* KO testes (Supplementary Figure S1D). However, HE staining and immunostaining of the testis sections of WT and *Ccdc92* KO mice revealed dramatic differences. Developing spermatids with flagella protruding into the tubular lumen were abundant in WT seminiferous tubules. By contrast, flagellated spermatozoa were almost absent within the *Ccdc92* KO tubular lumen (Figure 3A). The flagellum defects in *Ccdc92* KO seminiferous tubules were consistently evident from the immunostaining analysis of the sperm flagella using an acetylated tubulin antibody (Figure 3B).

Next, we performed periodic acid-Schiff (PAS) and hematoxylin staining to examine the developmental phases of spermatogenesis in WT and *Ccdc92* KO testes. Germ cells that were undergoing either proliferation or meiosis in *Ccdc92* KO seminiferous tubules were comparable to those in WT seminiferous tubules (Supplementary Figure S1E), indicating that CCDC92 is dispensable for the mitotic division of spermatogonia and meiosis of spermatocytes. We next examined the process of post-meiotic spermiogenesis, which can be grouped into four distinct phases: Golgi, cap, acrosome, and maturation (Figure 3C). Based on the changes in the acrosome and the nuclear morphology of the younger generation of spermatids, spermiogenesis is divided into 16 steps (Figure 3C). The first 12 steps of spermiogenesis are used to determine the subdivision of the cycle of the seminiferous tubules into 12 stages (stages I–XII) (Ahmed and de Rooij, 2009). The subsequent steps of spermiogenesis (steps 13–16) are found in the sequential stages (Kotaja, 2013). While the early steps of spermiogenesis appeared normal in *Ccdc92* KO spermatids (Supplementary Figure S1E), most significant defects were present in the later steps (Figure 3D). Elongating or elongated *Ccdc92* KO spermatids frequently exhibited malformed sperm heads with disorganized acrosomes (Figure 3D).

PNA staining was performed to further assess the status of acrosome biogenesis in the seminiferous tubules. In *Ccdc92* KO spermatids at the Golgi phase of spermiogenesis, a single PNA-labeled acrosomal granule was similarly formed at the center of the acrosomal vesicle (Supplementary Figure S1F). At the cap phase, the acrosomal granule in *Ccdc92* KO spermatids gradually enlarged to form a cap-like structure covering the nucleus, and then the cap-shaped acrosome further elongated and outlined the dorsal edge, similar to the acrosome changes in WT spermatids (Supplementary Figure S1F). These results indicate that CCDC92 is dispensable for early acrosome biogenesis. Consistently, the head abnormalities at the maturation phase were evident from the observations of malformed acrosomes and over-elongated nuclei in *Ccdc92* KO spermatids (Figure 3E), similar to those shown for the mature sperm in *Ccdc92* KO epididymides (Figure 2B, D, E, and H). These results strongly indicate that CCDC92 plays a pivotal role in spermiogenesis, especially in the later steps of sperm development.

### CCDC92 regulates the manchette for sperm head shaping

**Figure 4:**
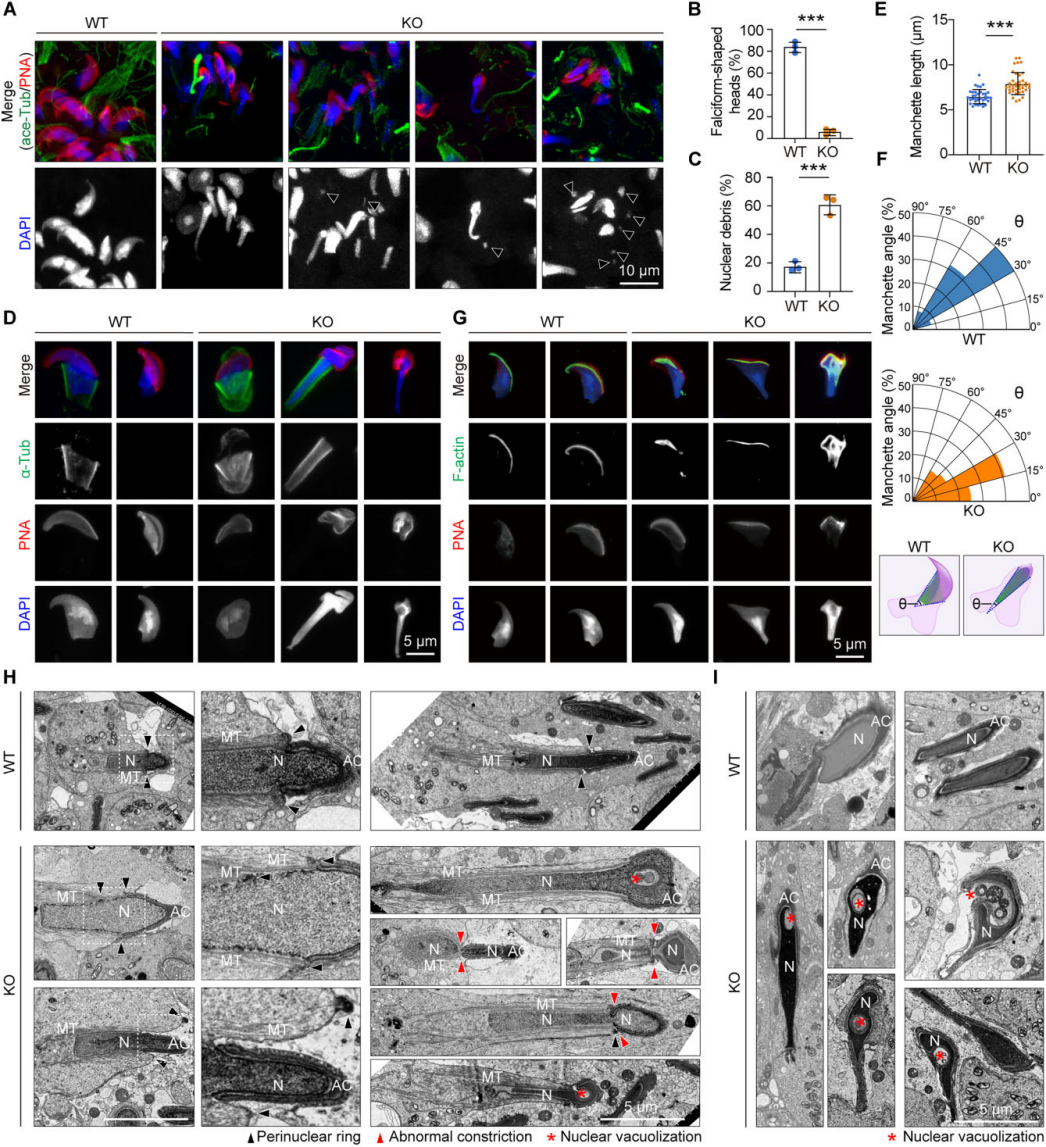
CCDC92 regulates the manchette for the sperm head shaping. (**A**) Immunofluorescence staining of WT and *Ccdc92* KO testis sections with the ace-Tub antibody, Alexa Fluor 568-conjugated PNA, and DAPI. The arrowheads indicate small nuclear debris. (**B** and **C**) Quantifications of the falciform-shaped heads (**B**) and nuclear debris (**C**) in WT and *Ccdc92* KO seminiferous tubules (*n* = 3 mice per genotype). (**D**–**F**) Testicular spermatids were isolated from the WT and *Ccdc92* KO testes. (**D**) Immunofluorescence staining with DAPI, Alexa Fluor 568-conjugated PNA, and the α-Tub antibody. (**E** and **F**) Quantifications of the manchette length and quarter circular polar plots of the manchette angle (θ) representing the manchette defects. The schematic diagrams illustrate the manchette angles in WT and *Ccdc92* KO spermatids. (**G**) Fluorescence staining of WT and *Ccdc92* KO testicular spermatids with DAPI, Alexa Fluor 568-conjugated PNA, and FITC-conjugated phalloidin (F-actin). (**H** and **I**) TEM images of the heads of elongating (**H**) and elongated (**I**) spermatids in WT and *Ccdc92* KO seminiferous tubules. Magnified images of the dashed boxed regions are shown on the right. Note that the anchoring of the acrosome (AC) to the nucleus (N) in elongating *Ccdc92* KO spermatids appears unaltered, whereas manchette microtubules (MT) are unconventionally over-elongated. Data are presented as mean ± SD. Unpaired two-tailed *t*-test was performed. ***P < 0.001.

We next investigated the effects of CCDC92 depletion on nuclear shaping. In the WT seminiferous tubules,∼84% of the elongated spermatids displayed typical falciform-shaped head morphology, whereas only 6% of the elongated *Ccdc92* KO spermatids had a curved falciform head (Figure 4A and B). In *Ccdc92* KO seminiferous tubules, most spermatids possessed an over-elongated or rod-like head, and small round nuclear debris was frequently observed (Figure 4A and C). These results suggest that CCDC92 is essential for sperm head shaping during spermiogenesis.

Regarding the essential role of the manchette in sperm head shaping (Lehti and Sironen, 2016; Wei and Yang, 2018; Hu et al., 2023), we explored whether the manchette was affected by the CCDC92 deficit. In elongating WT spermatids, the manchette microtubules assembled transiently in a mantle shape and disappeared when the head shaping was completed (Figure 4D). However, although the manchette microtubule mantle and the acrosome were generally formed at the beginning of head shaping, the manchette microtubules in elongating *Ccdc92* KO spermatids were unconventionally elongated, along with a portion of the nucleus remaining inside the manchette and elongating to a significant extent (Figure 4D and E). The manchette angles, defined by the manchette's outer planes, were measured to describe the manchette defects quantitatively (Figure 4F). The proportions of WT and *Ccdc92* KO spermatids with manchette angles ranging from 30° to 60° were 83% and 31%, respectively (Figure 4F). The majority (66%) of *Ccdc92* KO spermatids had manchette angles ranging from 0° to 30° (Figure 4F). Meanwhile, the acrosome became irregular and disorganized at the apical region of the head (Figure 4D). As for the actin filaments, no manchette-like labeling was observed in elongating WT spermatids (Figure 4G). The phalloidin signal was predominantly seen beneath the acrosome (Figure 4G), a narrow region between the acrosome and the nucleus. By contrast, the uniform distribution of actin filaments was disturbed in *Ccdc92* KO elongating spermatids. The phalloidin signal extended its range from the narrow subacrosomal region to the proximal nucleus and abnormally accumulated on the over-elongated nucleus (Figure 4G).

Next, TEM was performed to examine the ultrastructural changes. Consistent with the fluorescence results (Supplementary Figure S1F), the formation and anchoring of the acrosome to the nuclear membrane in round spermatids at the Golgi and cap phases were unaltered in *Ccdc92* KO mice (Supplementary Figure S2). However, the manchette perinuclear ring was mislocalized in elongating *Ccdc92* KO spermatids (Figure 4H). The manchette microtubules and their surrounding nucleus appeared strikingly over-elongated compared to those in WT spermatids (Figure 4H). Interestingly, abnormal constrictions at the perinuclear ring were frequently observed in *Ccdc92* KO spermatids at later development steps (Figure 4H), likely resulting in small nuclear debris observed in fluorescence images (Figure 4A). In elongated *Ccdc92* KO spermatids, detached acrosomes and nuclei with cytoplasmic materials protruding into the nucleus appeared to be dominant head defects (Figure 4I). These results showed that loss of CCDC92 caused severe disturbance to the manchette formation and function, which might lead to secondary acrosome detachment and nuclear abnormalities.

### CCDC92 interacts with CEP164 and IFT-B proteins

**Figure 5:**
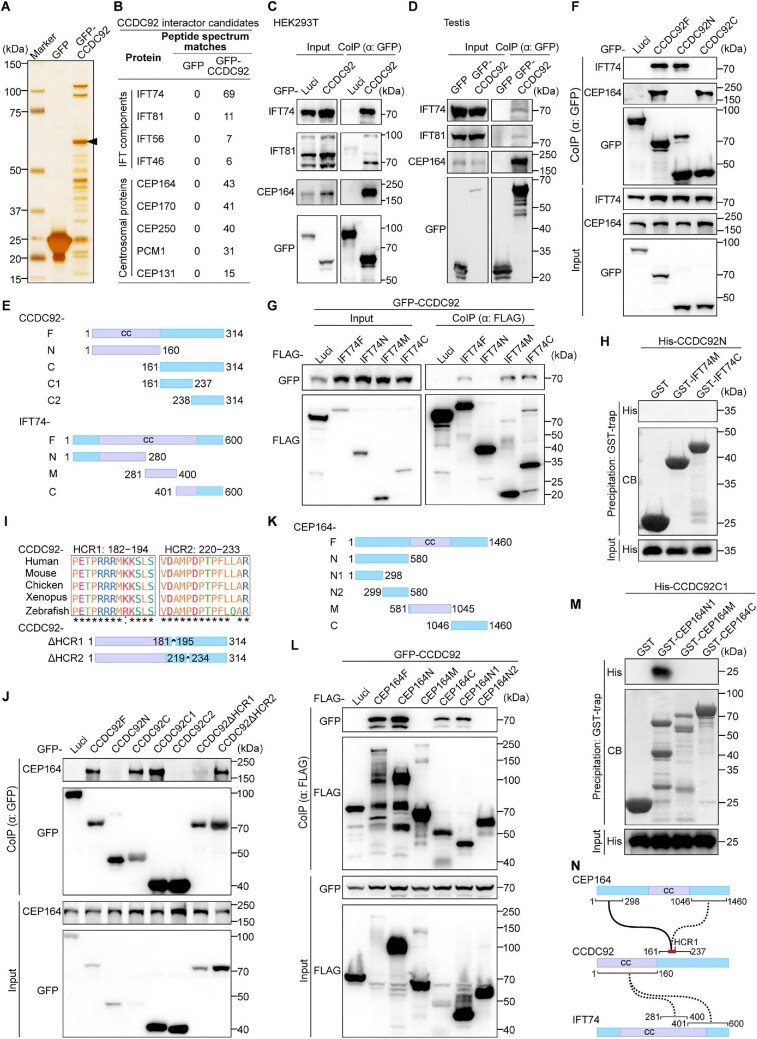
CCDC92 interacts with CEP164 and IFT-B proteins. (**A**) Silver staining of GFP and GFP-CCDC92 immunopurified proteins. The arrowhead indicates the GFP-CCDC92 band. (**B**) CCDC92 interactor candidates identified by mass spectrometry analysis. (**C** and **D**) CoIP analysis of exogenously expressed GFP-CCDC92 in HEK293T cells and testis lysates. GFP proteins were immunoprecipitated with GFP-Trap agarose beads and probed with IFT74, IFT81, CEP164, and GFP antibodies. Luci, luciferase. (**E**) Diagrams of CCDC92 and IFT74 truncated fragments. CCDC92 fragments were generated based on the protein structure prediction with AlphaFold. Cc, coiled-coil motif. (**F**) CoIP analysis of exogenously expressed GFP-tagged CCDC92 fragments in HEK293T cells. GFP proteins were immunoprecipitated with GFP-Trap agarose beads and probed with IFT74, CEP164, and GFP antibodies. (**G**) CoIP analysis of co-expressed GFP-CCDC92 and FLAG-tagged IFT74 fragments in HEK293T cells. FLAG-tagged proteins were immunoprecipitated with anti-FLAG beads and probed with FLAG and GFP antibodies. (**H**) GST pull-down assay using purified GST, GST-tagged IFT74M and IFT74C, and His-CCDC92N proteins. Coomassie blue (CB) staining showing the quantity of GST proteins. Co-purified proteins were probed with an anti-His antibody. (**I**) Identification of evolutionarily HCRs of CCDC92. Multiple CCDC92 protein sequences from typical vertebrates were analyzed with the Clustal Omega program. ‘*’ indicates fully conserved residue positions, and ‘:’ indicates strongly conserved residue positions. (**J**) CoIP analysis of exogenously expressed GFP-tagged CCDC92 fragments in HEK293T cells. (**K**) A diagram of CEP164 truncated fragments. (**L**) CoIP analysis of co-expressed GFP-CCDC92 and FLAG-tagged CEP164 fragments in HEK293T cells. (**M**) GST pull-down assay using purified GST, GST-tagged CEP164 fragments, and His-CCDC92C1 proteins. (**N**) A diagram showing the interactions of CCDC92 with IFT-B proteins and CEP164.

To dissect the molecular mechanism underlying CCDC92-associated male infertility, we performed co-immunoprecipitation (coIP) coupled with mass spectrometry to identify interacting partners of CCDC92. GFP-tagged CCDC92 was expressed in HEK293T cells, immunoprecipitated with GFP nanobody-coated agarose beads (Figure 5A), and subjected to on-bead digestion-based mass spectrometry analysis. After removing non-specific proteins by comparing hits from GFP and GFP-CCDC92 samples and selecting proteins with peptide spectrum matches < 5, a total of 365 candidate proteins were identified (Supplementary Table S1). CEP164, a previously reported interacting protein of CCDC92 (Chaki et al., 2012), was also on the list of candidate proteins, suggesting that the mass spectrometry result is reliable. Besides CEP164, multiple subunits of the IFT-B complex, including IFT74, IFT81, IFT56, and IFT46, were identified (Figure 5B). The interactions could be confirmed using antibodies against CEP164, IFT74, and IFT81 in coIP complexes from human HEK293T cells and mouse testis lysates (Figure 5C and D).

To characterize the interactions in more detail, we generated truncated CCDC92 fragments based on the protein structure predicted with AlphaFold (Figure 5E). Exogenous expression of GFP-tagged CCDC92 fragments in HEK293T cells followed by coIP showed that CCDC92 interacted with IFT components through its N-terminus. In contrast, its C-terminus mediated the binding to CEP164 (Figure 5F). Next, we mapped the binding sites of IFT74 with CCDC92. Immunoblotting revealed that the middle and C-terminus fragments of IFT74 (IFT74M and IFT74C) could associate with CCDC92 (Figure 5E and G). GST pull-down assay with purified proteins showed that IFT74M and IFT74C could not pull down the CCDC92 N-terminus protein (Figure 5H), indicating an indirect interaction between CCDC92 and IFT74.

As for CEP164, we further determined the binding regions in the C-terminus of CCDC92. Through multiple sequence alignments of CCDC92 protein sequences from representative vertebrate species, two highly conserved regions (HCR1: amino acids 182–194; HCR2: amino acids 220–233) were identified in the C-terminus (Figure 5I; Supplementary Figure S3). CoIP analysis revealed that deletion of HCR1 disrupted the binding of CCDDC92 to CEP164, whereas CCDC92 depleted HCR2 still interacted with CEP164 (Figure 5J), demonstrating that the highly conserved residues (PETPRRRMK/RKSLS) are necessary for its interaction with CEP164. We also generated truncated CEP164 fragments to investigate the interaction further (Figure 5K; Burke et al., 2014). The N-terminal mutants containing 1–298 amino acids and the C-terminal fragment spanning 1046–1460 amino acids firmly interacted with CCDC92 (Figure 5L). Interestingly, GST pull-down analysis showed that the purified CEP164 N-terminus fragment protein, but not the C-terminus fragment protein, was able to pull down the CCDC92 fragment protein (Figure 5M), indicating that CEP164 directly interacts with CCDC92 via its N-terminus. Collectively, these data demonstrate that CCDC92 can associate with IFT-B complex proteins and directly interact with CEP164 (Figure 5N).

### CCDC92 colocalizes with CEP164 and traffics with IFT along the cilium

**Figure 6:**
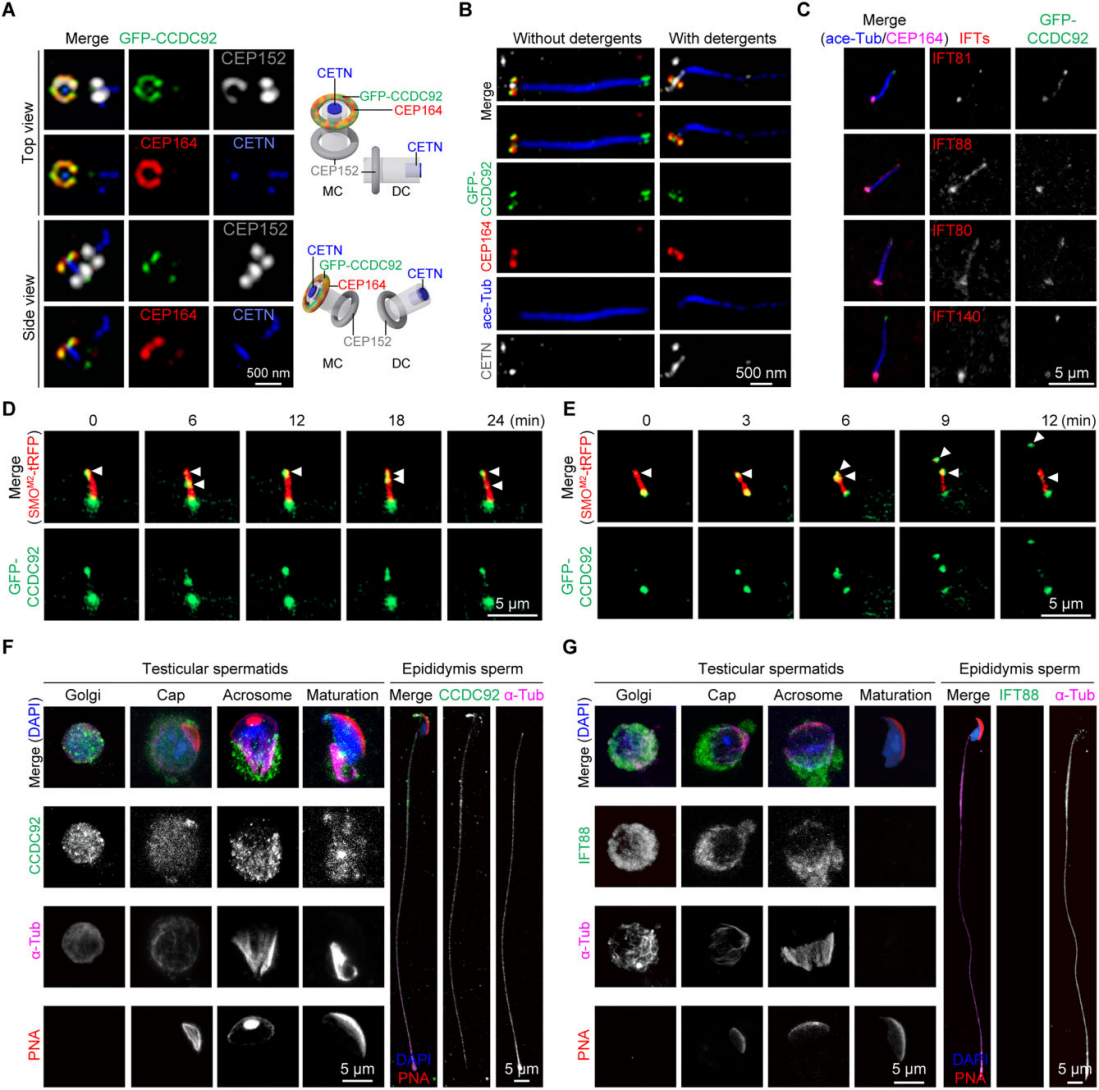
CCDC92 localizes to primary cilia and sperm manchette with IFT proteins. (**A**) Immunofluorescence staining of GFP-CCDC92 in human U2OS cells with CEP152, CEP164, and Centrin (CETN) antibodies, imaged with 3D-SIM. The diagrams illustrate the spatial distributions of the indicated proteins on the mother and daughter centrioles (MC and DC). (**B**) Immunofluorescence staining of GFP-CCDC92 in ciliated IMCD3 cells, fixed directly or pre-extracted with detergents, with ace-Tub, CEP164, and Centrin (CETN) antibodies, imaged with 3D-SIM. (**C**) Immunofluorescence staining of GFP-CCDC92 and IFT-B proteins in ciliated hTERT-RPE1 cells with ace-Tub, CEP164, and IFT antibodies. (**D** and **E**) Live-cell imaging of co-expressed GFP-CCDC92 and SMO^M2^-tRFP in hTERT-RPE1 cells serum-starved for 24 h. (**F** and **G**) Immunofluorescence staining of endogenous CCDC92 (**F**) and IFT88 (**G**) in spermatids at various steps of spermiogenesis and mature epididymis spermatozoa with α-tubulin (α-Tub) and CCDC92 or IFT88 antibodies, DAPI, and Alexa Fluor 568-conjugated PNA.

Regarding the interaction between CCDC92 and CEP164, we sought to investigate the precise localization of CCDC92 using 3D structured illumination microscopy (3D-SIM). CEP164 and CEP152 were utilized to indicate the distal end of the mother centriole and the proximal ends of both centrioles (Zhao et al., 2020, 2021). In non-ciliated human osteosarcoma U2OS cells, GFP-CCDC92 displayed a ring-shaped distribution at the distal end of the mother centriole, where it colocalized with CEP164 (Figure 6A). In ciliated mouse inner medulla collecting duct (IMCD3) cells, GFP-CCDC92 prominently concentrated and formed 1–2 bulges along the axoneme, mainly at the ciliary tip (Figure 6B). Upon disintegrating the membrane lipids with detergents, CCDC92 bulges were solubilized from cilia, but the centriolar CCDC92 was retained (Figure 6B).

Given the association of CCDC92 with IFT-B complex proteins, we carried out lentivirus-mediated GFP-CCDC92 expression in human retinal pigment epithelial hTERT-RPE1 cells and performed immunofluorescence staining of IFT proteins to examine their ciliary spatial relationships between CCDC92 and IFT proteins. As shown in Figure 6C, the IFT proteins that could associate with CCDC92 frequently accumulated at the ciliary bulges formed by GFP-CCDC92 in GFP-CCDC92-expressing cells. These data indicate that the deviant ciliary accumulations of IFT-B components are likely attributed to their association with CCDC92.

Based on these observations, we hypothesized that CCDC92 could traffic with IFT in the cilium and thus conducted live imaging to visualize the ciliary dynamics of CCDC92. Tag-RFP (tRFP)-tagged Smoothened (SMO) was used to mark the cilium. SMO is a ciliary transmembrane protein functioning as a critical signal transducer in the Hedgehog signaling pathway. The SMO W535L mutation, also known as the SMO^M2^ mutation, leads to constitutive ciliary SMO localization (Lu et al., 2015). hTERT-RPE1 cells expressing GFP-CCDC92 and SMO^M2^-tRFP were serum-starved to stimulate ciliogenesis. Live cell imaging revealed similar accumulations of GFP-CCDC92 at the ciliary base and tip (Figure 6D; Supplementary Video S2), a consistent distribution pattern with the immunostaining results. Indeed, the GFP-CCDC92 puncta displayed bidirectional movement at a slow speed along the axoneme (Figure 6D; Supplementary Video S2). Interestingly, we occasionally observed the release of ciliary vesicles from the ciliary distal end (Figure 6E; Supplementary Video S3). Together, these results demonstrate that CCDC92 traffics with IFT complex proteins within the cilium.

### CCDC92 and IFT proteins are distributed to the manchette in elongating spermatids

To further explore the localization of endogenous CCDC92, we generated a polyclonal antibody against mouse CCDC92. WT and *Ccdc92* KO spermatozoa were utilized to validate the antibody specificity for immunostaining. Compared with WT sperm cells, labeling of CCDC92 in the sperm head and flagellum was undetectable in *Ccdc92* KO spermatozoa (Supplementary Figure S4A). To verify whether the loss of the CCDC92 signal is due to sperm abnormalities, spermatozoa obtained from *Ccdc181* KO mice, which also produce malformed spermatozoa (Zi et al., 2024), were similarly subjected to immunostaining. In *Ccdc181* KO spermatozoa, the CCDC92 signal was found all over the short or coiled flagellum (Supplementary Figure S4A). The results indicate a specific reactivity of the CCDC92 antibody.

Next, we examined the cellular distribution of CCDC92 and IFT-B proteins in spermatids at various steps of spermiogenesis. At the Golgi and cap phases, CCDC92 was evenly distributed in the cytoplasm of the round spermatids. As the spermatid nucleated the manchette and progressed into the elongation phase, the CCDC92 signal was detected in the distal cytoplasmic lobe, where it colocalized with α-tubulin (Figure 6F), indicating that CCDC92 localizes to the manchette. As the manchette migrated downwards and came off the nucleus, CCDC92 was seen in the spermatid distal region and on the manchette. In addition, CCDC92 was observed at the apical tip and the ventral edge of the head (Figure 6F). In mature epididymis sperm, CCDC92 was steadily observed at the sperm flagellum and at the most apical tip of the sperm head (Figure 6F; Supplementary Figure S4A), a region known as the perforatorium that extends beyond the apical nucleus and creates the hook-like appearance of most murid spermatozoa (Protopapas et al., 2019).

Like CCDC92, IFT88 was distributed in the cytoplasm of the round spermatid (Figure 6G). As the acrosome extended over the nucleus with the manchette forming on the other side, IFT88 labeling became prominent on the manchette (Figure 6G). Consistent with the previous report (San Agustin et al., 2015), no IFT88 signal was observed in the spermatid at the maturation phase in seminiferous tubules or the epididymis sperm cells (Figure 6G). Additionally, IFT57 displayed a similar cellular distribution to IFT88 in spermatids at different steps (Supplementary Figure S4B). These data demonstrate that CCDC92 and IFT-B proteins are both distributed on the manchette in elongating spermatids.

### CCDC92 deficiency affects the proper flagellar distribution of MIPs

To evaluate the proteome-wide changes in developing *Ccdc92* KO spermatids, quantitative proteomics using the asymmetric track lossless (Astral) mass analyzer-based data-independent acquisition (DIA) was performed to identify the differentially expressed proteins (DEPs). Testes isolated from three WT and *Ccdc92* KO males were separately sampled for Astral DIA analysis. The number of proteins detected in each sample was comparable, ranging from 9230 to 9281 proteins (Supplementary Figure S5A). A total of 8662 proteins simultaneously detected in each sample were subjected to the principal component analysis (PCA) to examine intra- and inter-group proteomic variabilities. When ordinated by PCA, each genotype sample was separated from the other (Supplementary Figure S5B), indicating distinct proteomic changes between WT and *Ccdc92* KO testes.

**Figure 7:**
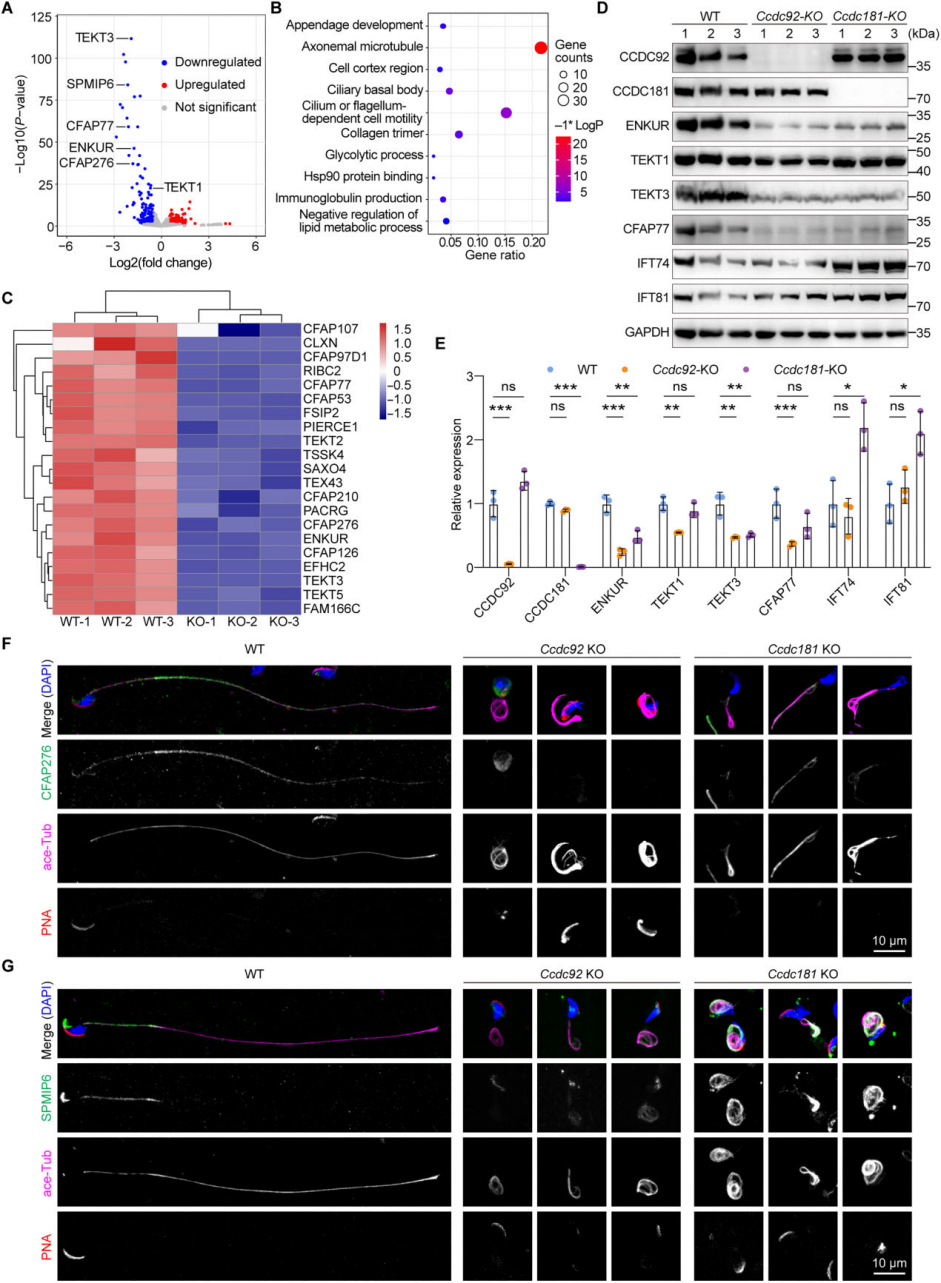
Quantitative proteomics reveals the functional association of CCDC92 with axonemal MIPs. (**A**) Volcano plot showing the DEPs between WT and *Ccdc92* KO testes identified with a cut-off for statistical significance (|log2(fold change)| > 0.584, *P* < 0.05). A total of 53 upregulated and 113 downregulated proteins were identified in *Ccdc92* KO testes. Proteins used for the following analysis are indicated. (**B**) GO enrichment analysis of DEPs identified in *Ccdc92* KO testes. The top 10 GO terms are presented. (**C**) Heatmap showing the DEPs in three *Ccdc92* KO testes (KO-1/2/3) and three WT testes (WT-1/2/3), enriched in GO terms of axonemal microtubule and cilium or flagellum-dependent cell motility. (**D** and **E**) Immunoblotting and quantifications showing the expression levels of the indicated protein in WT, *Ccdc92* KO, and *Ccdc181* KO testes. Testes isolated from three WT, *Ccdc92* KO, and *Ccdc181* KO mice were separately sampled and analyzed. GAPDH was used as a loading control. The band intensity of the indicated protein was normalized to the corresponding GAPDH band intensity. Data are presented as mean ± SD. Unpaired two-tailed *t*-test was performed. ns, not significant; *P < 0.05; **P < 0.01; ***P < 0.001. (**F** and **G**) Representative fluorescence images of WT, *Ccdc92* KO, and *Ccdc181* KO epididymis sperm cells. Mature spermatozoa released from the cauda epididymides of adult mice were stained with ace-Tub and CFAP276 (**F**) or SPMIP6 (**G**) antibodies, Alexa Fluor 568-conjugated PNA, and DAPI.

By setting a cut-off for statistical significance, a total of 166 DEPs were identified in *Ccdc92* KO testes, including 53 upregulated and 113 downregulated proteins (Figure 7A). We next performed Gene Ontology (GO) enrichment analysis to describe the functional roles of these DEPs, and the top 10 GO terms were visualized as a bubble plot (Figure 7B). Interestingly, GO enrichment analysis revealed that most DEPs were implicated in axonemal microtubule (GO: 0005879) and cilium or flagellum-dependent cell motility (GO: 0001539) (Figure 7B). The DEPs enriched in the GO terms of axonemal microtubule and cilium or flagellum-dependent cell motility were subjected to a supervised hierarchical clustering analysis across samples. As shown in Figure 7C, all 21 DEPs were decreased in *Ccdc92* KO testes and correctly clustered by genotype status. Strikingly, many of the DEPs identified are axonemal MIPs, such as ENKUR, CFAP107, and tektin family proteins (Figure 7C; Gui et al., 2021; Tai et al., 2023; Zhou et al., 2023). Immunoblotting confirmed a consistent decrease in protein levels of detected MIPs in *Ccdc92* KO testes. Meanwhile, the expression of IFT74 and IFT81 was not significantly affected compared to WT samples (Figure 7D and E). We next asked whether deformed flagellar axonemes caused the reduction in the MIP expression levels. To address this question, we employed *Ccdc181* KO mice to examine the effects. All the MIPs tested showed a significant reduction in *Ccdc92* KO testes, although we noted moderate reductions in *Ccdc181* KO mice (Figure 7D and E).

Next, we performed immunostaining to investigate the effects of CCDC92 depletion on the sperm flagellar MIPs. In WT spermatids, CFAP276 was distributed to the entire flagellum with a moderate accumulation at the principal piece. However, the flagellar CFAP276 signal in *Ccdc92* KO spermatids was barely detectable (Figure 7F). SPMIP6, another sperm MIP, was observed at the flagellar midpiece and the sperm head apical tip in WT spermatids, whereas its flagellar signal in *Ccdc92* KO spermatids was similarly decreased (Figure 7G). With the *Ccdc181* KO spermatids, we found that *Ccdc181* KO-associated sperm abnormalities did not lead to the reduction of flagellar MIPs (Figure 7F and G). Overall, these results suggest that the loss of CCDC92 in developing spermatids affects the flagellar distribution of axonemal MIPs.

## Discussion

In this study, we show that *Ccdc92* KO results in male infertility in mice. Further examination reveals that *Ccdc92* KO males are sterile due to severe abnormalities of the mature spermatids, including misshapen sperm heads and disorganized ‘9+2’ flagellar axoneme structure. CCDC92 can interact and traffic with IFT-B proteins. In developing spermatids, CCDC92 is localized to the manchette, where it regulates the manchette formation and function to participate in shaping the sperm head during spermiogenesis. More importantly, by the mass spectrometry-based quantitative proteomic analysis, we demonstrate a functional requirement of CCDC92 for proper flagellar distribution of axonemal MIPs.

Our results showed that CCDC92 depletion causes apparent abnormalities in the sperm head shape. Consistently, in *Ift88* KO male mice, the developing spermatids also display abnormally shaped nuclei (San Agustin et al., 2015). During spermiogenesis, sperm nuclear shaping is a very intricate and precise process that occurs in concert with the formation of the acrosome and manchette. Several studies suggest that the manchette influences nuclear shaping, participating in the morphogenesis of the spermatid head (Russell et al., 1991; Hu et al., 2023). As a temporary structure, the manchette forms when the nucleus deformation begins and disappears when the nuclear shaping is complete. We showed that CCDC92 and IFT proteins are distributed on the manchette during nuclear shaping, and analysis of *Ccdc92* KO spermatids revealed defective manchettes. The manchette defects occur earlier than other head abnormalities, such as acrosome dissociation and nuclear vacuolization. We thus consider these abnormalities as a secondary effect of the manchette defect. Our data strongly indicate an essential role of CCDC92 in the manchette during nuclear shaping. Still, detailed studies are needed to determine how CCDC92 regulates the manchette microtubule dynamics for shaping the sperm head.

The acrosome formation is another important event during spermiogenesis. The acrosome development starts from a single acrosomal vesicle derived from the Golgi apparatus and associates with the nuclear envelope (Khawar et al., 2019). As the acrosome vesicle expands over the nucleus, the inner acrosomal membrane is anchored to the nuclear envelope through an F-actin-containing structure named the acroplaxome (Zuo et al., 2024). Interestingly, the acroplaxome is also proposed to function in sperm head shaping (Wei and Yang, 2018). In *Ccdc92* KO mice, the anchoring of the acrosome to the nucleus in spermatids at early development steps was morphologically unaltered. As spermatids progressed into elongation, the actin filaments between the acrosome and the nucleus were disorganized and abnormally accumulated on the elongating nucleus. Moreover, the cytoplasmic materials were observed to protrude into the sperm nucleus. These findings indicate an essential role of CCDC92 in cytoplasm removal during spermiogenesis. Given the membrane-associating property of CCDC92 (Figure 6B), we hypothesize that CCDC92 may regulate vesicular trafficking to participate in this process.

In addition to the sperm head abnormalities mentioned above, the majority of *Ccdc92* KO flagella were morphologically malformed. It has been well demonstrated that IFT transports ciliary proteins to build cilia and is also required for sperm tail development (Ishikawa and Marshall, 2017; Zhang, 2021; Zhao et al., 2023). Consistent with the previous report (San Agustin et al., 2015), the flagellar staining of IFT proteins in mature spermatids released from the epididymis is much weaker than that in developing spermatids isolated from the testis, indicative of a lower turnover of flagellar components in mature spermatids. Similarly, many sperm heads of *Ift88* KO spermatids are abnormally shaped, and the flagellum assembly is also impaired (San Agustin et al., 2015). Although multiple IFT proteins have been known to be indispensable for spermiogenesis (Zhang, 2021), detailed mechanisms remain largely unknown. We found that CCDC92 could interact and traffic with IFT along the ciliary axoneme, providing a potential mechanism for understanding IFT during spermiogenesis. In addition, quantitative proteomic analysis reveals a significant reduction in the expression levels of MIPs in *Ccdc92* KO testes (Figure 7). However, the expression of some MIPs was also comparatively affected in *Ccdc181* KO mice. The decrease in MIP expression may be a secondary effect of the deformed axoneme, which requires further exploration.

We found that depletion of CCDC92 impaired the formation of sperm flagellum but didn't cause noticeable developmental abnormalities in mice. As a modified motile cilium, the sperm flagellum can power sperm cell movement, consistent with the function of motile cilia in motility. However, primary cilia are present in most vertebrate cells as a cellular sensory organelle essential for the development and function of many tissues (Anvarian et al., 2019). Although exogenously expressed GFP-CCDC92 colocalizes with CEP164 and traffics along the axoneme with the IFT machinery, we failed to detect the ciliary distribution of endogenous CCDC92 in primary ciliated hTERT-RPE1 or IMCD3 cells. A recent study using a different *Ccdc92* KO mouse model showed that the KO mice developed reduced obesity and increased insulin sensitivity under high-fat diet conditions, indicative of a function of CCDC92 in the white adipose tissue (Ren et al., 2023). Moreover, podocyte-specific deletion of *Ccdc92* can ameliorate podocyte injury and proteinuria in diabetic mice (Zuo et al., 2024). In addition to CEP164 and IFT-B proteins, many other molecules were identified as potential CCDC92 interactors in our mass spectrometry analysis. Considering the importance of primary cilia in multiple tissues (Anvarian et al., 2019; Bai et al., 2022; Scamfer et al., 2022), CCDC92 may cooperate with its specific partners and function in other tissue types through primary cilia.

To date, a significant number of coiled-coil domain-containing (CCDC) proteins have been identified as essential factors for ciliogenesis and the male flagellum (Priyanka and Yenugu, 2021; Zhao et al., 2022; Liu et al., 2023). Mutations of many CCDC proteins are associated with a set of human-inherited diseases referred to as ciliopathies (Reiter and Leroux, 2017). In this study, we demonstrate that CCDC92 is essential for proper spermiogenesis in mice. Protein sequence alignment of mouse and human CCDC92 reveals that the amino acid identity is 87%. Considering that mouse and human spermatogenesis processes are highly conserved, CCDC92 is probably required for male fertility in humans, albeit a lack of clinical verifications in MMAF patients.

## Materials and methods

### Plasmids

Full-length mouse *Ccdc92* (NM_001418046), *Ift74* (NM_026319), *Cfap77* (NM-001166705), *Cfap276* (NM_029314), and *Spmip6* (NM_001048005) were amplified from a mouse cDNA library by PCR. Full-length human *CEP164* (NM_014956) was obtained from the DNASU Plasmid Repository (Arizona State University). Full-length and relative fragments were PCR-amplified and subcloned into the recombinational donor vector pDONR221 to generate entry clones (11789100, Thermo Fisher). LR recombination reactions between entry clones and desired gateway destination vectors (Kit # 1000000211, Addgene) (Wall et al., 2014) were performed to generate the expression constructs (11791100, Thermo Fisher). To generate the bacterial expression constructs, relative fragments were PCR-amplified and subcloned into pGEX-4T-1 or pET-28a to create the GST- or His- constructs. All constructs were verified via Sanger sequencing analysis. All the primers used are listed in Supplementary Table S2.

### Fluorescence microscopy

Cells on cover glasses were fixed with 4% paraformaldehyde (PFA) in phosphate-buffered saline (PBS) for 15 min at room temperature, rinsed with PBS, extracted with 0.5% Triton X-100 in PBS for 15 min, rinsed with PBS, and then blocked with 4% bovine serum albumin (BSA) in Tris-buffered saline with 0.1% Tween 20 (TBST) for 1 h. Primary antibodies were diluted in the blocking buffer and incubated with cells at 4°C overnight. Samples were then washed three times with the blocking buffer for 5 min each. The second antibodies diluted in blocking buffer were applied to the samples and incubated for 1 h at room temperature. Alexa Fluor 568-conjugated Lectin PNA (L32458, Thermo Fisher), FITC-labeled phalloidin (RM02836, Abclonal), and 4′,6-diamidino-2-phenylindole (DAPI) were used to mark the acrosome, F-actin, and DNA, respectively. Super-resolution images were obtained with a 3D structured illumination microscope at 125-nm intervals and further processed with the SoftWoRx software (DeltaVision OMX SR imaging system). The confocal images were acquired using a Leica TCS SP8 confocal platform equipped with an HCX Plan Apo 63×/1.40 oil immersion objective, with each scanned line averaged four times. Optical sections were captured at 0.5-μm intervals, and z-stack images were processed with maximum intensity projections (Leica Microsystems). All the antibodies used are listed in Supplementary Table S3.

### Animals

The *Ccdc92* KO mouse model (NM-KO-210284) was obtained from Shanghai Model Organisms Center. The KO founder mice were generated using the CRISPR–Cas9 system with dual sgRNAs (sgRNA #1: 5′-CAGGGCAACTGGGAGAATAAagg-3′; sgRNA #2: 5′-CAGGCGATTCATATTCTAGGtgg-3′) to delete the Exon 4 of the mouse *Ccdc92* gene (Transcript ID: ENSMUST00000036206.13). The resulting heterozygous mutated mice had a 1385-bp deletion and a 1-bp insertion into this genome region. *Ccdc92* KO mice were genotyped using 2×Taq Plus Master Mix Ⅱ (P213, Vazyme). The primers used for genotyping are listed in Supplementary Table S2. The *Ccdc181* KO mouse model (T032025) was generated as previously reported (Zi et al., 2024). All the mouse work was approved by the Animal Experimental Ethics Committee of Shandong Normal University (AEECSDNU2024080).

### Assessment of sperm quality

The assessment of mouse epididymal sperm motility was performed as described previously (Song et al., 2022). Briefly, the cauda epididymis was dissected from 8-week-old male mice. Spermatozoa were released from the cauda epididymis into 1 ml PBS for 10 min at 37°C. Then, 4 μl of the suspension was placed into the glass counting chamber, and the sperm motility was recorded with the CASA image system (IVOS II, Hamilton Thorne). For epididymal sperm count, the dissected cauda epididymis was cut into pieces with a pair of fine-pointed scissors, and spermatozoa were fully released into 1 ml PBS for 30 min at 37°C. The sperm suspension was further diluted, and the sperm cell concentration was determined using a hemocytometer.

### Preparation of testicular spermatogenic cells

Adult male mice (at least 8 weeks old) were sacrificed by cervical dislocation. The testes were surgically dissected and digested with 1 mg/ml collagenase I (HY-O0004, MCE) and 1 mg/ml hyaluronidase (HY-107910, MCE) in PBS for 10 min at 37 °C with continuous shaking at 100 rpm. The cells were further separated by gentle pipetting and filtered through 70-μm cell strainers (CSS013070, Guangzhou Jet Bio-Filtration). Then, dissociated testicular cells were precipitated by centrifugation at 500*× g* for 10 min and resuspended in 3 ml PBS. For immunostaining, 100 μl of the cell suspension was placed on a poly-lysine-coated slide for 15 min and subjected to fixation with 4% PFA in PBS for 15 min.

### Statistical analysis

All experiments were biologically repeated at least three times. For each experiment, 3–5 mice for each genotype were used. One representative picture from 3–5 mice for each genotype was presented for immunostaining. The relative intensity of protein bands on immunoblots was measured using ImageJ (National Institutes of Health). The quantitative results were presented as mean ± standard deviation (SD) unless specified in the figure legend. Statistical analyses were conducted using GraphPad Prism. Unpaired two-tailed Student's *t*-tests were used for comparison between the two groups. Differences were considered significant when *P*-value was <0.05.

## Supplementary Material

mjaf022_Supplemental_Files

## Notes

**Author contributions:** T.S. and H.Z. designed the study. Y.L., X.Z., and Q. Lyu performed experiments. Q. Li, Y.L., X.Z., J.Z., H.Z., and T.S. analyzed the results and wrote the manuscript. Q. Li, H.Y., Y.W., Q.C., B.K., and S.N. assisted with experiments. T.S. and H.Z. supervised and directed the research. All authors discussed the results and commented on the manuscript.
