# Supplementary material for: Intraflagellar transport-associated CCDC92 is required for spermiogenesis and male fertility in mice
Source: J Mol Cell Biol. 2025 Aug 5;17(5):mjaf022. doi: 10.1093/jmcb/mjaf022 (PMC12802938; doi:10.1093/jmcb/mjaf022)
Supplement: mjaf022_Supplemental_Files [file mjaf022_supplemental_files.zip › Supplementary Material 1R.pdf]

Supplementary Material For

Intraflagellar transport-associated CCDC92 is required for spermiogenesis and male fertility in mice

Yue Lu[1, #], Xirui Zi[1, #], Qian Lyu[1, #], Qingchao Li[1], Hanxiao Yin[2], Yinghao Wang[1], Qijun Chen[1], Bingkun Kang[1], Shanshan Nai[1], Jun Zhou[1, 2], Huijie Zhao[1, *], and Ting Song[1, *]

* Correspondence: 623056@sdnu.edu.cn (T.S.) or huijiezhao@sdnu.edu.cn (H.Z.)

This PDF file includes:

Supplemental figures (S1 to S5);

Supplemental tables (S1 to S3);

Supplemental methods;

Supplemental video legends.

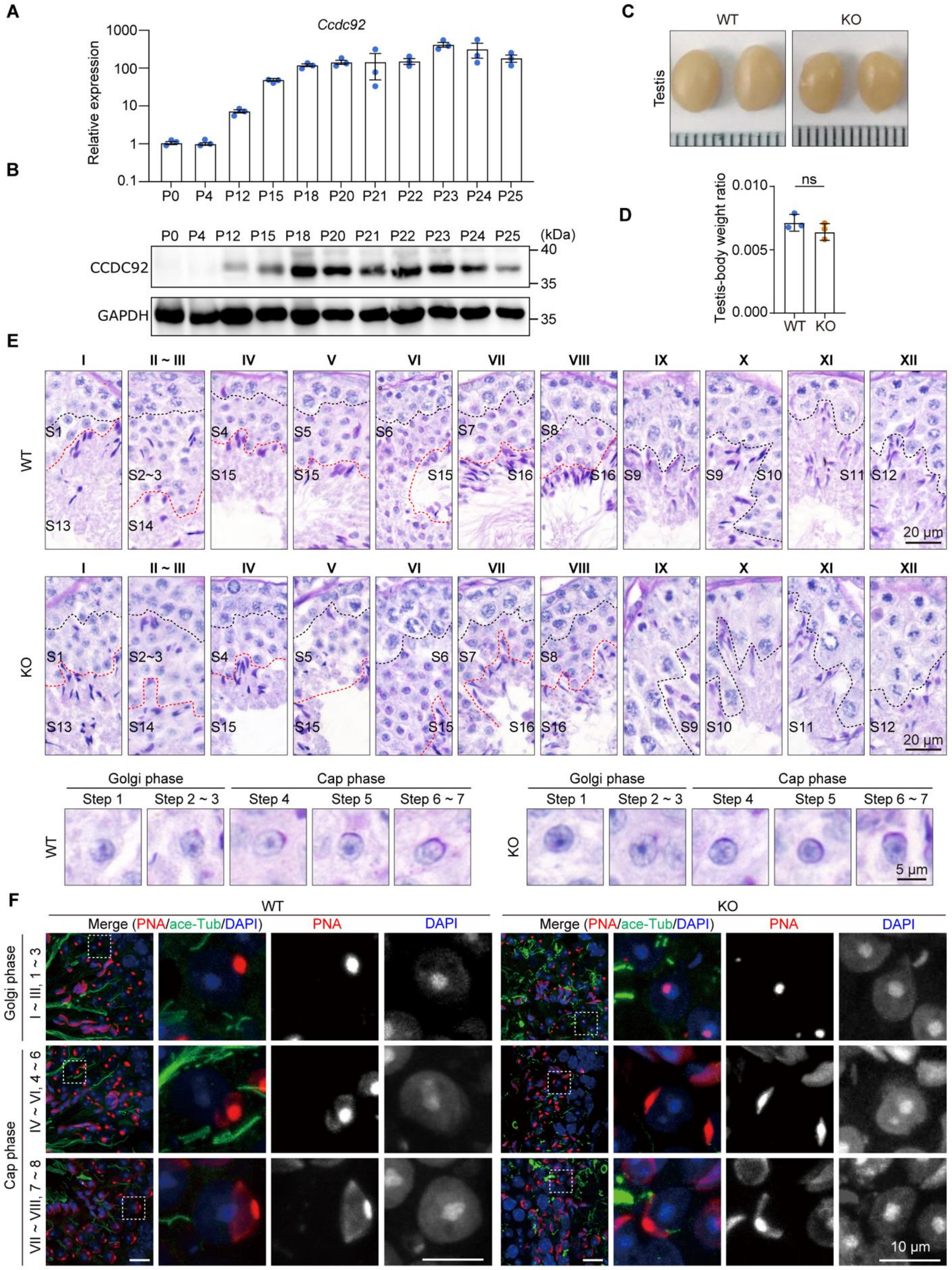

A
Ccdc92
Relative expression
1000
100
10
1
0.1
P0
P4
P12
P15
P18
P20
P21
P22
P23
P24
P25
B
(kDa)
CCDC92
GAPDH
40
35
35
C
WT
KO
Testis
D
Testis-body weight ratio
ns
0.010
0.005
0.000
WT
KO
E
I
II ~ III
IV
V
VI
VII
VIII
IX
X
XI
XII
WT
KO
S1
S2~3
S4
S5
S6
S7
S8
S9
S10
S11
S12
S13
S14
S15
S16
20 μm
Golgi phase
Cap phase
Step 1
Step 2 ~ 3
Step 4
Step 5
Step 6 ~ 7
5 μm
F
Merge (PNA/ace-Tub/DAPI)
PNA
DAPI
Golgi phase
I ~ III, 1 ~ 3
Cap phase
IV ~ VI, 4 ~ 6
VII ~ VIII, 7 ~ 8
10 μm

Figure S1 CCDC92 is essential for proper spermiogenesis in seminiferous tubules.

(**A**) Real-time PCR analysis showed the expression of *Ccdc92* in testes isolated from mice of the indicated age. *Ccdc92* expression was normalized using the corresponding *Gapdh* as the reference gene and baseline 1 (P0) as the reference sample (△△CT method). Data are from three independent biological repeats and presented on a logarithmic scale ($\log_{10}$) as mean ± SEM. (**B**) Immunoblotting showed the expression of CCDC92 in testes isolated from mice of the indicated age. GAPDH was used as a loading control. (**C**) Representative images of WT and *Ccdc92* KO testes from eight-week-old mice. (**D**) Testis-to-body weight ratio of adult WT and *Ccdc92* KO mice (n = 3 mice per genotype). Data are presented as mean ± SD. Unpaired two-tailed *t*-test was performed. ns, not significant. (**E**) Periodic acid-Schiff (PAS) and hematoxylin staining of WT and *Ccdc92* KO testis sections. Testicular regions containing spermatids at different development steps were outlined with black and red dashed lines. No obvious defects were observed in the early steps of spermiogenesis in *Ccdc92* KO spermatids. (**F**) Fluorescence images of seminiferous tubules containing spermatids at Golgi and cap phases in WT and *Ccdc92* KO testis sections. Sections were stained with an acetylated α-tubulin (ace-Tub) antibody, Alexa Fluor 568-conjugated PNA, and DAPI. Magnified images of the dashed boxed regions are shown on the right.

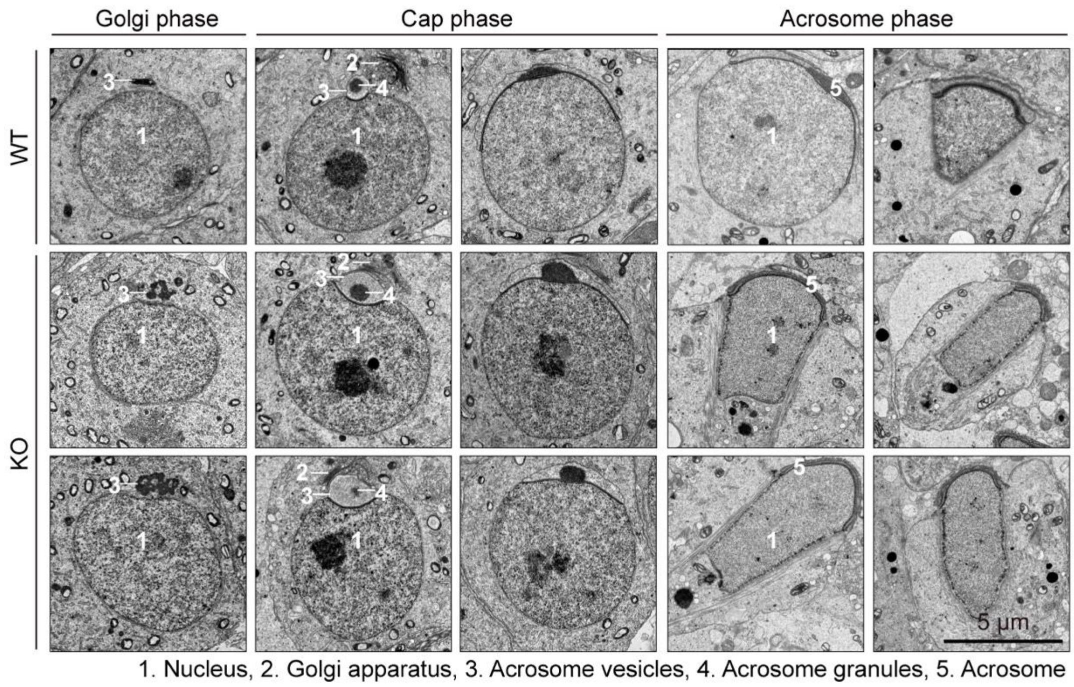

Figure S2 CCDC92 is dispensable for the early steps of spermatid differentiation.

Transmission electron microscopy images of the nuclei of spermatids at early development phases (Golgi, cap, and acrosome phases) in WT and *Ccdc92* KO seminiferous tubules. Note that the formation and anchoring of the acrosome to the nucleus in *Ccdc92 KO* spermatids appeared unaltered.

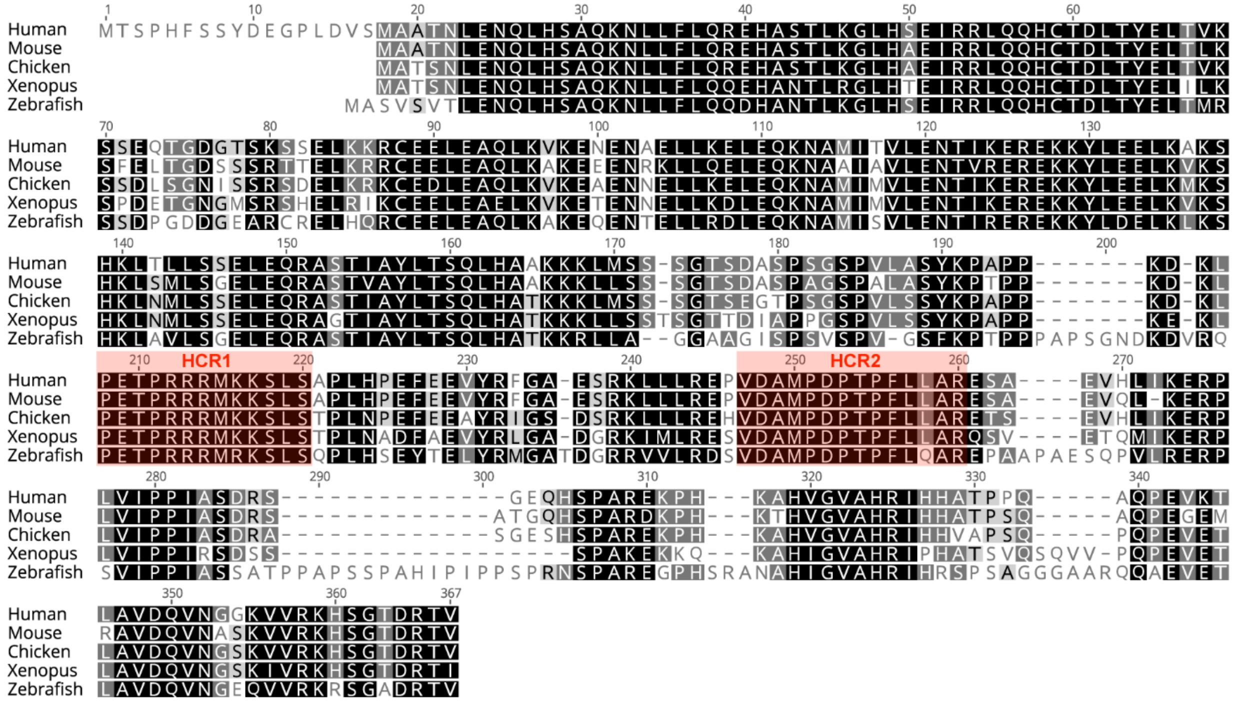

Figure S3 Identification of evolutionarily highly conserved regions (HCRs) of CCDC92. Multiple CCDC92 protein sequences from typical vertebrates were analyzed with the MAFFT program. The protein sequences of CCDC92 orthologs (Human, NP_001291886; Mouse, NP_001404975; Chicken, XP_040504302; Xenopus, NP_001072794; Zebrafish, NP_001032794) were used for analysis. A black background indicates fully conserved residue positions, and a grey background indicates strongly conserved residue positions. Two evolutionarily highly conserved regions (HCR1 and HCR2) are highlighted.

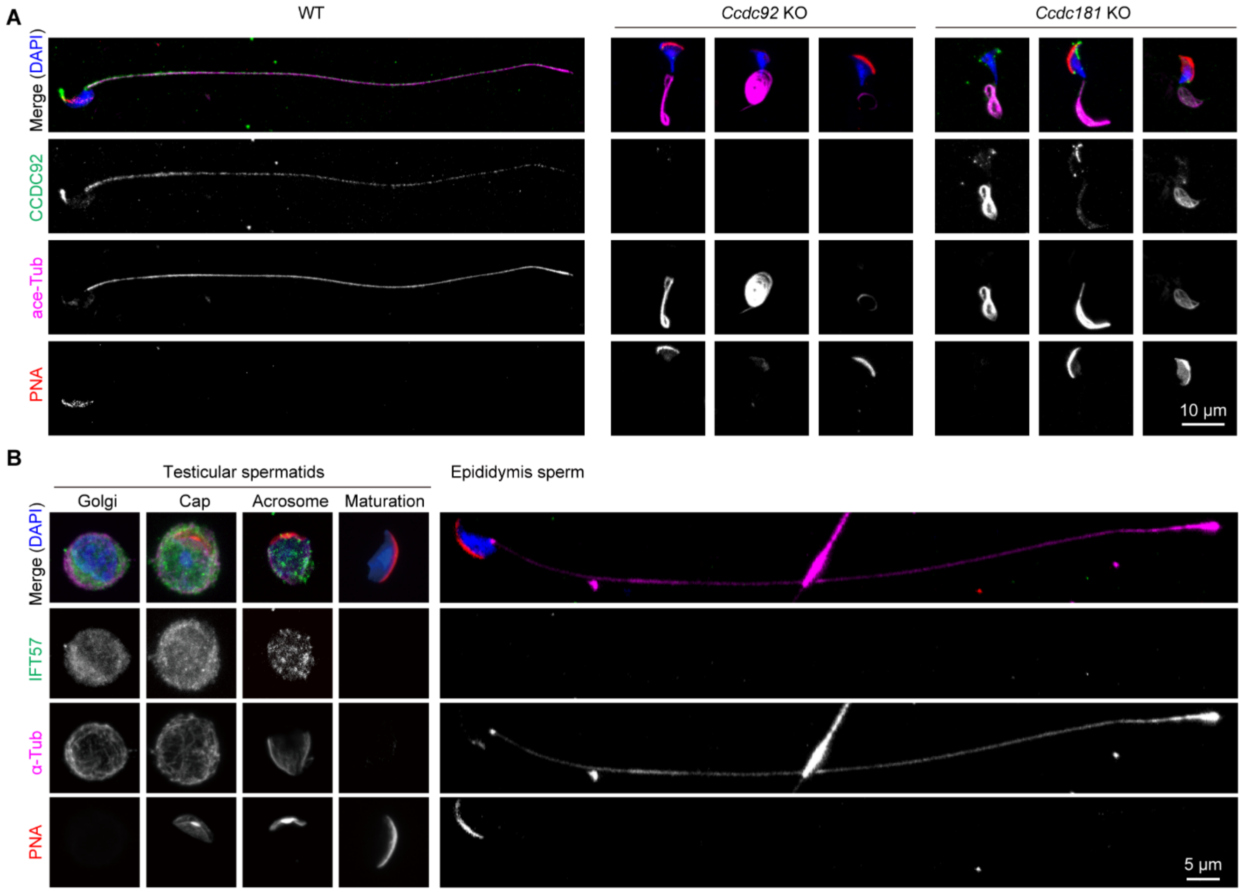

Figure S4 CCDC92 localizes to the sperm manchette with IFT proteins.

(**A**) Immunofluorescence images of WT, *Ccdc92* KO, and *Ccdc181* KO epididymis sperm cells. Mature spermatozoa released from the cauda epididymides of adult mice were stained with acetylated α-tubulin (ace-Tub) and CCDC92 antibodies, Alexa Fluor 568 conjugated PNA, and DAPI. (**B**) Fluorescence images of endogenous IFT57 in spermatids at various steps of spermiogenesis and mature epididymis spermatozoa. Spermatids isolated from the testis and spermatozoa released from the epididymis were immunostained with α-tubulin (α-Tub) and IFT57 antibodies. The nuclei were stained with DAPI, and the acrosomes were stained with Alexa Fluor 568-conjugated PNA.

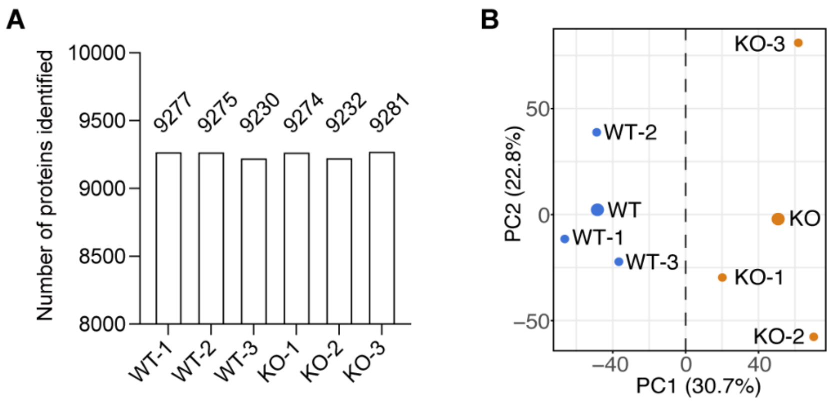

Figure S5 Quantitative proteomics reveals remarkable changes in *Ccdc92* KO testes.

(**A**) The number of proteins identified from each sample using the asymmetric track lossless (Astral) mass analyzer-based data-independent acquisition (DIA). (**B**) Principal component analysis (PCA) of proteome data. Note that WT samples were clearly separated from *Ccdc92* KO samples.

Supplementary Table 1: List of CCDC92 interactor candidates

Accession	Description	Peptide spectrum matches (PSM)	
		GFP	GFP-CCDC92
Q99460	26S proteasome non-ATPase regulatory subunit 1 OS=Homo sapiens GN=PSMD1 PE=1 SV=2	0	493
Q13200	26S proteasome non-ATPase regulatory subunit 2 OS=Homo sapiens GN=PSMD2 PE=1 SV=3	0	379
O00231	26S proteasome non-ATPase regulatory subunit 11 OS=Homo sapiens GN=PSMD11 PE=1 SV=3	0	376
P17980	26S protease regulatory subunit 6A OS=Homo sapiens GN=PSMC3 PE=1 SV=3	0	348
P43686	26S protease regulatory subunit 6B OS=Homo sapiens GN=PSMC4 PE=1 SV=2	0	311
O00232	26S proteasome non-ATPase regulatory subunit 12 OS=Homo sapiens GN=PSMD12 PE=1 SV=3	0	287
O43242	26S proteasome non-ATPase regulatory subunit 3 OS=Homo sapiens GN=PSMD3 PE=1 SV=2	0	281
P35998	26S protease regulatory subunit 7 OS=Homo sapiens GN=PSMC2 PE=1 SV=3	0	274
P62258	14-3-3 protein epsilon OS=Homo sapiens GN=YWHAE PE=1 SV=1	0	265
P62191	26S protease regulatory subunit 4 OS=Homo sapiens GN=PSMC1 PE=1 SV=1	0	262
F5H2Z7	Coiled-coil domain-containing protein 92 (Fragment) OS=Homo sapiens GN=CCDC92 PE=1 SV=1	0	246
P54886	Delta-1-pyrroline-5-carboxylate synthase OS=Homo sapiens GN=ALDH18A1 PE=1 SV=2	0	244
A0A087X2I1	26S protease regulatory subunit 10B OS=Homo sapiens GN=PSMC6 PE=1 SV=1	0	243
Q9UNM6	26S proteasome non-ATPase regulatory subunit 13 OS=Homo sapiens GN=PSMD13 PE=1 SV=2	0	178
Q15008	26S proteasome non-ATPase regulatory subunit 6 OS=Homo sapiens GN=PSMD6 PE=1 SV=1	0	174
P55036	26S proteasome non-ATPase regulatory subunit 4 OS=Homo sapiens GN=PSMD4 PE=1 SV=1	0	168
P51665	26S proteasome non-ATPase regulatory subunit 7 OS=Homo sapiens GN=PSMD7 PE=1 SV=2	0	163
P61981	14-3-3 protein gamma OS=Homo sapiens GN=YWHAG PE=1 SV=2	0	122
O00487	26S proteasome non-ATPase regulatory subunit 14 OS=Homo sapiens GN=PSMD14 PE=1 SV=1	0	118
O14818	Proteasome subunit alpha type-7 OS=Homo sapiens GN=PSMA7 PE=1 SV=1	0	118
Q5VWC4	26S proteasome non-ATPase regulatory subunit 4 OS=Homo sapiens GN=PSMD4 PE=1 SV=1	0	115
P25786	Proteasome subunit alpha type-1 OS=Homo sapiens GN=PSMA1 PE=1 SV=1	0	113

P31946	14-3-3 protein beta/alpha OS=Homo sapiens GN=YWHAB PE=1 SV=3	0	112
Q04917	14-3-3 protein eta OS=Homo sapiens GN=YWHAH PE=1 SV=4	0	111
P04350	Tubulin beta-4A chain OS=Homo sapiens GN=TUBB4A PE=1 SV=2	0	98
P20618	Proteasome subunit beta type-1 OS=Homo sapiens GN=PSMB1 PE=1 SV=2	0	96
Q71U36	Tubulin alpha-1A chain OS=Homo sapiens GN=TUBA1A PE=1 SV=1	0	89
P28070	Proteasome subunit beta type-4 OS=Homo sapiens GN=PSMB4 PE=1 SV=4	0	85
P63104	14-3-3 protein zeta/delta OS=Homo sapiens GN=YWHAZ PE=1 SV=1	0	85
P49720	Proteasome subunit beta type-3 OS=Homo sapiens GN=PSMB3 PE=1 SV=2	0	84
P60900	Proteasome subunit alpha type-6 OS=Homo sapiens GN=PSMA6 PE=1 SV=1	0	84
P28066	Proteasome subunit alpha type-5 OS=Homo sapiens GN=PSMA5 PE=1 SV=3	0	81
P48556	26S proteasome non-ATPase regulatory subunit 8 OS=Homo sapiens GN=PSMD8 PE=1 SV=2	0	80
Q5LJA5	Ubiquitin carboxyl-terminal hydrolase OS=Homo sapiens GN=UCHL5 PE=1 SV=1	0	77
Q99436	Proteasome subunit beta type-7 OS=Homo sapiens GN=PSMB7 PE=1 SV=1	0	77
Q9Y5K5	Ubiquitin carboxyl-terminal hydrolase isozyme L5 OS=Homo sapiens GN=UCHL5 PE=1 SV=3	0	76
P54652	Heat shock-related 70 kDa protein 2 OS=Homo sapiens GN=HSPA2 PE=1 SV=1	0	75
O75832	26S proteasome non-ATPase regulatory subunit 10 OS=Homo sapiens GN=PSMD10 PE=1 SV=1	0	71
Q96LB3	Intraflagellar transport protein 74 homolog OS=Homo sapiens GN=IFT74 PE=1 SV=1	0	69
P68366	Tubulin alpha-4A chain OS=Homo sapiens GN=TUBA4A PE=1 SV=1	0	68
P25788	Proteasome subunit alpha type-3 OS=Homo sapiens GN=PSMA3 PE=1 SV=2	0	64
P25789	Proteasome subunit alpha type-4 OS=Homo sapiens GN=PSMA4 PE=1 SV=1	0	61
M0QZM1	Heterogeneous nuclear ribonucleoprotein M (Fragment) OS=Homo sapiens GN=HNRNPM PE=1 SV=1	0	59
P49721	Proteasome subunit beta type-2 OS=Homo sapiens GN=PSMB2 PE=1 SV=1	0	57
Q15386	Ubiquitin-protein ligase E3C OS=Homo sapiens GN=UBE3C PE=1 SV=3	0	54
Q14997	Proteasome activator complex subunit 4 OS=Homo sapiens GN=PSME4 PE=1 SV=2	0	53
P12235	ADP/ATP translocase 1 OS=Homo sapiens GN=SLC25A4 PE=1 SV=4	0	53
A0A024RA52	Proteasome subunit alpha type OS=Homo sapiens GN=PSMA2 PE=1 SV=1	0	52
Q16186	Proteasomal ubiquitin receptor ADRM1 OS=Homo sapiens GN=ADRM1 PE=1 SV=2	0	52
O95714	E3 ubiquitin-protein ligase HERC2 OS=Homo sapiens GN=HERC2 PE=1 SV=2	0	51

Q05086	Ubiquitin-protein ligase E3A OS=Homo sapiens GN=UBE3A PE=1 SV=4	0	47
A2A3N6	Putative PIP5K1A and PSMD4-like protein OS=Homo sapiens GN=PIPSL PE=5 SV=1	0	47
Q9UPV0	Centrosomal protein of 164 kDa OS=Homo sapiens GN=CEP164 PE=1 SV=3	0	43
Q92616	Translational activator GCN1 OS=Homo sapiens GN=GCN1L1 PE=1 SV=6	0	43
P28072	Proteasome subunit beta type-6 OS=Homo sapiens GN=PSMB6 PE=1 SV=4	0	42
Q5SW79	Centrosomal protein of 170 kDa OS=Homo sapiens GN=CEP170 PE=1 SV=1	0	41
Q9BV73	Centrosome-associated protein CEP250 OS=Homo sapiens GN=CEP250 PE=1 SV=2	0	40
P34932	Heat shock 70 kDa protein 4 OS=Homo sapiens GN=HSPA4 PE=1 SV=4	0	37
E7ETA6	Pericentriolar material 1 protein OS=Homo sapiens GN=PCM1 PE=1 SV=1	0	31
H0Y2W2	ATPase family AAA domain-containing protein 3A (Fragment) OS=Homo sapiens GN=ATAD3A PE=1 SV=1	0	28
Q9BQS8	FYVE and coiled-coil domain-containing protein 1 OS=Homo sapiens GN=FYCO1 PE=1 SV=3	0	26
J3KNL6	Protein transport protein Sec16A OS=Homo sapiens GN=SEC16A PE=1 SV=1	0	26
O95757	Heat shock 70 kDa protein 4L OS=Homo sapiens GN=HSPA4L PE=1 SV=3	0	25
P46087	Probable 28S rRNA (cytosine(4447)-C(5))-methyltransferase OS=Homo sapiens GN=NOP2 PE=1 SV=2	0	24
Q8NB90	Spermatogenesis-associated protein 5 OS=Homo sapiens GN=SPATA5 PE=1 SV=3	0	24
A0A087WVV1	Proteasome subunit beta type-2 OS=Homo sapiens GN=PSMB2 PE=1 SV=1	0	23
Q9Y230	RuvB-like 2 OS=Homo sapiens GN=RUVBL2 PE=1 SV=3	0	23
Q14204	Cytoplasmic dynein 1 heavy chain 1 OS=Homo sapiens GN=DYNC1H1 PE=1 SV=5	0	22
Q6P2Q9	Pre-mRNA-processing-splicing factor 8 OS=Homo sapiens GN=PRPF8 PE=1 SV=2	0	22
Q5W0B1	RING finger protein 219 OS=Homo sapiens GN=RNF219 PE=1 SV=1	0	21
Q7Z6Z7	E3 ubiquitin-protein ligase HUWE1 OS=Homo sapiens GN=HUWE1 PE=1 SV=3	0	21
Q9NVI1	Fanconi anemia group I protein OS=Homo sapiens GN=FANCI PE=1 SV=4	0	21
P68032	Actin, alpha cardiac muscle 1 OS=Homo sapiens GN=ACTC1 PE=1 SV=1	0	21
P17706	Tyrosine-protein phosphatase non-receptor type 2 OS=Homo sapiens GN=PTPN2 PE=1 SV=2	0	20
P46821	Microtubule-associated protein 1B OS=Homo sapiens GN=MAP1B PE=1 SV=2	0	20
O95071	E3 ubiquitin-protein ligase UBR5 OS=Homo sapiens GN=UBR5 PE=1 SV=2	0	20
H3BVG0	Nuclear pore complex protein Nup93 OS=Homo sapiens GN=NUP93 PE=1 SV=1	0	19
C9J406	MICOS complex subunit MIC60 OS=Homo sapiens GN=IMMT PE=1 SV=1	0	18

Q5H9R7	Serine/threonine-protein phosphatase 6 regulatory subunit 3 OS=Homo sapiens GN=PPP6R3 PE=1 SV=2	0	18
O00567	Nucleolar protein 56 OS=Homo sapiens GN=NOP56 PE=1 SV=4	0	17
P30876	DNA-directed RNA polymerase II subunit RPB2 OS=Homo sapiens GN=POLR2B PE=1 SV=1	0	17
P55072	Transitional endoplasmic reticulum ATPase OS=Homo sapiens GN=VCP PE=1 SV=4	0	17
Q93008	Probable ubiquitin carboxyl-terminal hydrolase FAF-X OS=Homo sapiens GN=USP9X PE=1 SV=3	0	17
O14744	Protein arginine N-methyltransferase 5 OS=Homo sapiens GN=PRMT5 PE=1 SV=4	0	17
Q9BRP4	Proteasomal ATPase-associated factor 1 OS=Homo sapiens GN=PAAF1 PE=1 SV=2	0	17
Q2KHR3	Glutamine and serine-rich protein 1 OS=Homo sapiens GN=QSER1 PE=1 SV=3	0	16
A0A087WY61	Nuclear mitotic apparatus protein 1 OS=Homo sapiens GN=NUMA1 PE=1 SV=1	0	16
Q9BZE4	Nucleolar GTP-binding protein 1 OS=Homo sapiens GN=GTPBP4 PE=1 SV=3	0	16
P61353	60S ribosomal protein L27 OS=Homo sapiens GN=RPL27 PE=1 SV=2	0	16
Q9C0C7	Activating molecule in BECN1-regulated autophagy protein 1 OS=Homo sapiens GN=AMBRA1 PE=1 SV=2	0	15
Q9UPN4	Centrosomal protein of 131 kDa OS=Homo sapiens GN=CEP131 PE=1 SV=3	0	15
O14980	Exportin-1 OS=Homo sapiens GN=XPO1 PE=1 SV=1	0	15
Q92621	Nuclear pore complex protein Nup205 OS=Homo sapiens GN=NUP205 PE=1 SV=3	0	15
Q8WVY7	Ubiquitin-like domain-containing CTD phosphatase 1 OS=Homo sapiens GN=UBLCP1 PE=1 SV=2	0	15
Q96EY1	DnaJ homolog subfamily A member 3, mitochondrial OS=Homo sapiens GN=DNAJA3 PE=1 SV=2	0	14
Q8NG31	Protein CASC5 OS=Homo sapiens GN=CASC5 PE=1 SV=3	0	14
Q86V48	Leucine zipper protein 1 OS=Homo sapiens GN=LUZP1 PE=1 SV=2	0	14
Q6NSI4	Uncharacterized protein CXorf57 OS=Homo sapiens GN=CXorf57 PE=1 SV=2	0	14
Q9HCE1	Putative helicase MOV-10 OS=Homo sapiens GN=MOV10 PE=1 SV=2	0	14
G3V4C1	Heterogeneous nuclear ribonucleoproteins C1/C2 OS=Homo sapiens GN=HNRNPC PE=1 SV=1	0	14
P62888	60S ribosomal protein L30 OS=Homo sapiens GN=RPL30 PE=1 SV=2	0	14
O60763	General vesicular transport factor p115 OS=Homo sapiens GN=USO1 PE=1 SV=2	0	13
Q92900	Regulator of nonsense transcripts 1 OS=Homo sapiens GN=UPF1 PE=1 SV=2	0	13
Q9NVP1	ATP-dependent RNA helicase DDX18 OS=Homo sapiens GN=DDX18 PE=1 SV=2	0	13
Q14974	Importin subunit beta-1 OS=Homo sapiens GN=KPNB1 PE=1 SV=2	0	13
Q9H3G5	Probable serine carboxypeptidase CPVL OS=Homo sapiens GN=CPVL PE=1 SV=2	0	13

Q14684	Ribosomal RNA processing protein 1 homolog B OS=Homo sapiens GN=RRP1B PE=1 SV=3	0	13
P04637	Cellular tumor antigen p53 OS=Homo sapiens GN=TP53 PE=1 SV=4	0	13
P62249	40S ribosomal protein S16 OS=Homo sapiens GN=RPS16 PE=1 SV=2	0	13
Q68DQ2	Very large A-kinase anchor protein OS=Homo sapiens GN=CRYBG3 PE=1 SV=3	0	12
P53621	Coatomer subunit alpha OS=Homo sapiens GN=COPA PE=1 SV=2	0	12
Q15366	Poly(rC)-binding protein 2 OS=Homo sapiens GN=PCBP2 PE=1 SV=1	0	12
O43823	A-kinase anchor protein 8 OS=Homo sapiens GN=AKAP8 PE=1 SV=1	0	12
Q96JM3	Chromosome alignment-maintaining phosphoprotein 1 OS=Homo sapiens GN=CHAMP1 PE=1 SV=2	0	12
Q9P2J5	Leucine--tRNA ligase, cytoplasmic OS=Homo sapiens GN=LARS PE=1 SV=2	0	12
O00311	Cell division cycle 7-related protein kinase OS=Homo sapiens GN=CDC7 PE=1 SV=1	0	12
Q02978	Mitochondrial 2-oxoglutarate/malate carrier protein OS=Homo sapiens GN=SLC25A11 PE=1 SV=3	0	12
Q9Y2W1	Thyroid hormone receptor-associated protein 3 OS=Homo sapiens GN=THRAP3 PE=1 SV=2	0	12
Q9Y2X3	Nucleolar protein 58 OS=Homo sapiens GN=NOP58 PE=1 SV=1	0	11
Q7Z4H7	HAUS augmin-like complex subunit 6 OS=Homo sapiens GN=HAUS6 PE=1 SV=2	0	11
Q8WYA0	Intraflagellar transport protein 81 homolog OS=Homo sapiens GN=IFT81 PE=1 SV=1	0	11
Q7L2E3	Putative ATP-dependent RNA helicase DHX30 OS=Homo sapiens GN=DHX30 PE=1 SV=1	0	11
Q9UPY3	Endoribonuclease Dicer OS=Homo sapiens GN=DICER1 PE=1 SV=3	0	11
Q6ZRV2	Protein FAM83H OS=Homo sapiens GN=FAM83H PE=1 SV=3	0	11
O00165	HCLS1-associated protein X-1 OS=Homo sapiens GN=HAX1 PE=1 SV=2	0	11
P51587	Breast cancer type 2 susceptibility protein OS=Homo sapiens GN=BRCA2 PE=1 SV=3	0	11
A0A0C4DGG9	Chromodomain-helicase-DNA-binding protein 4 OS=Homo sapiens GN=CHD4 PE=1 SV=1	0	11
H0YFY6	Nuclear mitotic apparatus protein 1 (Fragment) OS=Homo sapiens GN=NUMA1 PE=1 SV=1	0	11
Q96A65	Exocyst complex component 4 OS=Homo sapiens GN=EXOC4 PE=1 SV=1	0	11
Q5T4S7	E3 ubiquitin-protein ligase UBR4 OS=Homo sapiens GN=UBR4 PE=1 SV=1	0	11
K7ELC2	40S ribosomal protein S15 OS=Homo sapiens GN=RPS15 PE=1 SV=1	0	11
A0A087WXU3	Extended synaptotagmin-2 OS=Homo sapiens GN=ESYT2 PE=1 SV=1	0	10
Q96TA2	ATP-dependent zinc metalloprotease YME1L1 OS=Homo sapiens GN=YME1L1 PE=1 SV=2	0	10
P10644	cAMP-dependent protein kinase type I-alpha regulatory subunit OS=Homo sapiens GN=PRKAR1A PE=1 SV=1	0	10

Q9H078	Caseinolytic peptidase B protein homolog OS=Homo sapiens GN=CLPB PE=1 SV=1	0	10
P42696	RNA-binding protein 34 OS=Homo sapiens GN=RBM34 PE=1 SV=2	0	10
Q16576	Histone-binding protein RBBP7 OS=Homo sapiens GN=RBBP7 PE=1 SV=1	0	10
O00443	Phosphatidylinositol 4-phosphate 3-kinase C2 domain-containing subunit alpha OS=Homo sapiens GN=PIK3C2A PE=1 SV=2	0	10
P61289	Proteasome activator complex subunit 3 OS=Homo sapiens GN=PSME3 PE=1 SV=1	0	10
Q7L576	Cytoplasmic FMR1-interacting protein 1 OS=Homo sapiens GN=CYFIP1 PE=1 SV=1	0	10
Q9UPN7	Serine/threonine-protein phosphatase 6 regulatory subunit 1 OS=Homo sapiens GN=PPP6R1 PE=1 SV=5	0	10
O75190	DnaJ homolog subfamily B member 6 OS=Homo sapiens GN=DNAJB6 PE=1 SV=2	0	10
E9PN76	RING finger protein 214 OS=Homo sapiens GN=RNF214 PE=1 SV=1	0	10
P11802	Cyclin-dependent kinase 4 OS=Homo sapiens GN=CDK4 PE=1 SV=2	0	10
Q15149	Plectin OS=Homo sapiens GN=PLEC PE=1 SV=3	0	10
O60343	TBC1 domain family member 4 OS=Homo sapiens GN=TBC1D4 PE=1 SV=2	0	9
O95793	Double-stranded RNA-binding protein Staufen homolog 1 OS=Homo sapiens GN=STAU1 PE=1 SV=2	0	9
P14373	Zinc finger protein RFP OS=Homo sapiens GN=TRIM27 PE=1 SV=1	0	9
Q9Y5V3	Melanoma-associated antigen D1 OS=Homo sapiens GN=MAGED1 PE=1 SV=3	0	9
P60842	Eukaryotic initiation factor 4A-I OS=Homo sapiens GN=EIF4A1 PE=1 SV=1	0	9
P04843	Dolichyl-diphosphooligosaccharide--protein glycosyltransferase subunit 1 OS=Homo sapiens GN=RPN1 PE=1 SV=1	0	9
Q14126	Desmoglein-2 OS=Homo sapiens GN=DSG2 PE=1 SV=2	0	9
Q8WUM0	Nuclear pore complex protein Nup133 OS=Homo sapiens GN=NUP133 PE=1 SV=2	0	9
Q13823	Nucleolar GTP-binding protein 2 OS=Homo sapiens GN=GNL2 PE=1 SV=1	0	9
Q96JN8	Neuralized-like protein 4 OS=Homo sapiens GN=NEURL4 PE=1 SV=2	0	9
I3L2X7	Centrosomal protein of 131 kDa (Fragment) OS=Homo sapiens GN=CEP131 PE=1 SV=1	0	9
Q5JTH9	RRP12-like protein OS=Homo sapiens GN=RRP12 PE=1 SV=2	0	9
Q13439	Golgin subfamily A member 4 OS=Homo sapiens GN=GOLGA4 PE=1 SV=1	0	9
O43379	WD repeat-containing protein 62 OS=Homo sapiens GN=WDR62 PE=1 SV=4	0	9
Q12788	Transducin beta-like protein 3 OS=Homo sapiens GN=TBL3 PE=1 SV=2	0	9
P13674	Prolyl 4-hydroxylase subunit alpha-1 OS=Homo sapiens GN=P4HA1 PE=1 SV=2	0	9

Q96GX5	Serine/threonine-protein kinase greatwall OS=Homo sapiens GN=MASTL PE=1 SV=1	0	9
P33993	DNA replication licensing factor MCM7 OS=Homo sapiens GN=MCM7 PE=1 SV=4	0	9
Q8TED0	U3 small nucleolar RNA-associated protein 15 homolog OS=Homo sapiens GN=UTP15 PE=1 SV=3	0	9
Q06787	Fragile X mental retardation protein 1 OS=Homo sapiens GN=FMR1 PE=1 SV=1	0	9
Q9UJC3	Protein Hook homolog 1 OS=Homo sapiens GN=HOOK1 PE=1 SV=2	0	9
Q13615	Myotubularin-related protein 3 OS=Homo sapiens GN=MTMR3 PE=1 SV=3	0	9
E7EV93	Pericentriolar material 1 protein (Fragment) OS=Homo sapiens GN=PCM1 PE=1 SV=1	0	9
P54727	UV excision repair protein RAD23 homolog B OS=Homo sapiens GN=RAD23B PE=1 SV=1	0	9
Q9Y4W2	Ribosomal biogenesis protein LAS1L OS=Homo sapiens GN=LAS1L PE=1 SV=2	0	8
Q9Y2X9	Zinc finger protein 281 OS=Homo sapiens GN=ZNF281 PE=1 SV=1	0	8
O95347	Structural maintenance of chromosomes protein 2 OS=Homo sapiens GN=SMC2 PE=1 SV=2	0	8
J3KPF3	4F2 cell-surface antigen heavy chain OS=Homo sapiens GN=SLC3A2 PE=1 SV=1	0	8
A0A087X295	WD repeat-containing protein 6 OS=Homo sapiens GN=WDR6 PE=1 SV=1	0	8
A0A087WWK8	IQ motif and SEC7 domain-containing protein 1 OS=Homo sapiens GN=IQSEC1 PE=1 SV=1	0	8
O75691	Small subunit processome component 20 homolog OS=Homo sapiens GN=UTP20 PE=1 SV=3	0	8
P57740	Nuclear pore complex protein Nup107 OS=Homo sapiens GN=NUP107 PE=1 SV=1	0	8
Q9H9B4	Sideroflexin-1 OS=Homo sapiens GN=SFXN1 PE=1 SV=4	0	8
G3V0J0	Fragile X mental retardation 1, isoform CRA_e OS=Homo sapiens GN=FMR1 PE=1 SV=1	0	8
P53618	Coatomer subunit beta OS=Homo sapiens GN=COPB1 PE=1 SV=3	0	8
E7EVJ5	Cytoplasmic FMR1-interacting protein 2 OS=Homo sapiens GN=CYFIP2 PE=1 SV=1	0	8
Q14697	Neutral alpha-glucosidase AB OS=Homo sapiens GN=GANAB PE=1 SV=3	0	8
A0A087WV86	Protein aurora borealis OS=Homo sapiens GN=BORA PE=1 SV=1	0	8
Q6PKG0	La-related protein 1 OS=Homo sapiens GN=LARP1 PE=1 SV=2	0	8
Q13011	Delta(3,5)-Delta(2,4)-dienoyl-CoA isomerase, mitochondrial OS=Homo sapiens GN=ECH1 PE=1 SV=2	0	8
P14625	Endoplasmin OS=Homo sapiens GN=HSP90B1 PE=1 SV=1	0	8
E7ERS3	Zinc finger CCCH domain-containing protein 18 OS=Homo sapiens GN=ZC3H18 PE=1 SV=1	0	8
P35221	Catenin alpha-1 OS=Homo sapiens GN=CTNNA1 PE=1 SV=1	0	7
Q9H6R4	Nucleolar protein 6 OS=Homo sapiens GN=NOL6 PE=1 SV=2	0	7

Q9UQR1	Zinc finger protein 148 OS=Homo sapiens GN=ZNF148 PE=1 SV=2	0	7
P49792	E3 SUMO-protein ligase RanBP2 OS=Homo sapiens GN=RANBP2 PE=1 SV=2	0	7
Q6R327	Rapamycin-insensitive companion of mTOR OS=Homo sapiens GN=RICTOR PE=1 SV=1	0	7
Q96N67	Dedicator of cytokinesis protein 7 OS=Homo sapiens GN=DOCK7 PE=1 SV=4	0	7
Q5QJE6	Deoxynucleotidyltransferase terminal-interacting protein 2 OS=Homo sapiens GN=DNTTIP2 PE=1 SV=2	0	7
Q8TDD1	ATP-dependent RNA helicase DDX54 OS=Homo sapiens GN=DDX54 PE=1 SV=2	0	7
B0YIW6	Archain 1, isoform CRA_a OS=Homo sapiens GN=ARCN1 PE=1 SV=1	0	7
Q8WVX9	Fatty acyl-CoA reductase 1 OS=Homo sapiens GN=FAR1 PE=1 SV=1	0	7
Q86Y56	Dynein assembly factor 5, axonemal OS=Homo sapiens GN=DNAAF5 PE=1 SV=4	0	7
Q7Z3U7	Protein MON2 homolog OS=Homo sapiens GN=MON2 PE=1 SV=3	0	7
Q9GZR7	ATP-dependent RNA helicase DDX24 OS=Homo sapiens GN=DDX24 PE=1 SV=1	0	7
P54709	Sodium/potassium-transporting ATPase subunit beta-3 OS=Homo sapiens GN=ATP1B3 PE=1 SV=1	0	7
C9JFV4	Proline-, glutamic acid- and leucine-rich protein 1 OS=Homo sapiens GN=PELP1 PE=1 SV=2	0	7
P42166	Lamina-associated polypeptide 2, isoform alpha OS=Homo sapiens GN=TMPO PE=1 SV=2	0	7
Q96DT7	Zinc finger and BTB domain-containing protein 10 OS=Homo sapiens GN=ZBTB10 PE=1 SV=2	0	7
Q14008	Cytoskeleton-associated protein 5 OS=Homo sapiens GN=CKAP5 PE=1 SV=3	0	7
P46060	Ran GTPase-activating protein 1 OS=Homo sapiens GN=RANGAP1 PE=1 SV=1	0	7
P38432	Coilin OS=Homo sapiens GN=COIL PE=1 SV=1	0	7
M0R2Z9	SURP and G-patch domain-containing protein 2 OS=Homo sapiens GN=SUGP2 PE=1 SV=1	0	7
O60287	Nucleolar pre-ribosomal-associated protein 1 OS=Homo sapiens GN=URB1 PE=1 SV=4	0	7
Q13895	Bystin OS=Homo sapiens GN=BYSL PE=1 SV=3	0	7
Q99832	T-complex protein 1 subunit eta OS=Homo sapiens GN=CCT7 PE=1 SV=2	0	7
Q13435	Splicing factor 3B subunit 2 OS=Homo sapiens GN=SF3B2 PE=1 SV=2	0	7
A0AVF1	Intraflagellar transport protein 56 OS=Homo sapiens GN=TTC26 PE=2 SV=1	0	7
Q96D09	G-protein coupled receptor-associated sorting protein 2 OS=Homo sapiens GN=GPRASP2 PE=1 SV=1	0	7
O15226	NF-kappa-B-repressing factor OS=Homo sapiens GN=NKRF PE=1 SV=2	0	7
Q8WWM7	Ataxin-2-like protein OS=Homo sapiens GN=ATXN2L PE=1 SV=2	0	7
O14545	TRAF-type zinc finger domain-containing protein 1 OS=Homo sapiens GN=TRAFD1 PE=1 SV=1	0	7

Q15050	Ribosome biogenesis regulatory protein homolog OS=Homo sapiens GN=RRS1 PE=1 SV=2	0	7
Q9NTJ3	Structural maintenance of chromosomes protein 4 OS=Homo sapiens GN=SMC4 PE=1 SV=2	0	7
P23246	Splicing factor, proline- and glutamine-rich OS=Homo sapiens GN=SFPQ PE=1 SV=2	0	7
Q9UMZ2	Synergin gamma OS=Homo sapiens GN=SYNRG PE=1 SV=2	0	7
P82650	28S ribosomal protein S22, mitochondrial OS=Homo sapiens GN=MRPS22 PE=1 SV=1	0	7
P02786	Transferrin receptor protein 1 OS=Homo sapiens GN=TFRC PE=1 SV=2	0	7
P46013	Antigen KI-67 OS=Homo sapiens GN=MKI67 PE=1 SV=2	0	7
P42766	60S ribosomal protein L35 OS=Homo sapiens GN=RPL35 PE=1 SV=2	0	7
Q86YM7	Homer protein homolog 1 OS=Homo sapiens GN=HOMER1 PE=1 SV=2	0	7
C9IZQ1	Translocon-associated protein subunit alpha OS=Homo sapiens GN=SSR1 PE=1 SV=1	0	7
Q9GZL7	Ribosome biogenesis protein WDR12 OS=Homo sapiens GN=WDR12 PE=1 SV=2	0	7
Q9BT25	HAUS augmin-like complex subunit 8 OS=Homo sapiens GN=HAUS8 PE=1 SV=3	0	6
Q5SRD1	Putative mitochondrial import inner membrane translocase subunit Tim23B OS=Homo sapiens GN=TIMM23B PE=5 SV=2	0	6
Q7Z2W4	Zinc finger CCCH-type antiviral protein 1 OS=Homo sapiens GN=ZC3HAV1 PE=1 SV=3	0	6
P08708	40S ribosomal protein S17 OS=Homo sapiens GN=RPS17 PE=1 SV=2	0	6
H3BM74	NEDD8 ultimate buster 1 OS=Homo sapiens GN=NUB1 PE=1 SV=1	0	6
Q9H8H0	Nucleolar protein 11 OS=Homo sapiens GN=NOL11 PE=1 SV=1	0	6
A0A087X2H1	E3 ubiquitin-protein ligase HECTD1 OS=Homo sapiens GN=HECTD1 PE=1 SV=1	0	6
Q96CS2	HAUS augmin-like complex subunit 1 OS=Homo sapiens GN=HAUS1 PE=1 SV=1	0	6
Q92522	Histone H1x OS=Homo sapiens GN=H1FX PE=1 SV=1	0	6
F8W930	Insulin-like growth factor 2 mRNA-binding protein 2 OS=Homo sapiens GN=IGF2BP2 PE=1 SV=1	0	6
P33991	DNA replication licensing factor MCM4 OS=Homo sapiens GN=MCM4 PE=1 SV=5	0	6
P78316	Nucleolar protein 14 OS=Homo sapiens GN=NOP14 PE=1 SV=3	0	6
Q9P035	Very-long-chain (3R)-3-hydroxyacyl-CoA dehydratase 3 OS=Homo sapiens GN=HACD3 PE=1 SV=2	0	6
Q99661	Kinesin-like protein KIF2C OS=Homo sapiens GN=KIF2C PE=1 SV=2	0	6
P49756	RNA-binding protein 25 OS=Homo sapiens GN=RBM25 PE=1 SV=3	0	6
Q8IUF1	COBW domain-containing protein 2 OS=Homo sapiens GN=CBWD2 PE=1 SV=1	0	6

Q9Y5K6	CD2-associated protein OS=Homo sapiens GN=CD2AP PE=1 SV=1	0	6
O95373	Importin-7 OS=Homo sapiens GN=IPO7 PE=1 SV=1	0	6
P04406	Glyceraldehyde-3-phosphate dehydrogenase OS=Homo sapiens GN=GAPDH PE=1 SV=3	0	6
Q92973	Transportin-1 OS=Homo sapiens GN=TNPO1 PE=1 SV=2	0	6
P19022	Cadherin-2 OS=Homo sapiens GN=CDH2 PE=1 SV=4	0	6
Q9BQ70	Transcription factor 25 OS=Homo sapiens GN=TCF25 PE=1 SV=1	0	6
Q8WVM8	Sec1 family domain-containing protein 1 OS=Homo sapiens GN=SCFD1 PE=1 SV=4	0	6
P42704	Leucine-rich PPR motif-containing protein, mitochondrial OS=Homo sapiens GN=LRPPRC PE=1 SV=3	0	6
Q8IX07	Zinc finger protein ZFPM1 OS=Homo sapiens GN=ZFPM1 PE=1 SV=2	0	6
Q5VWN6	Protein FAM208B OS=Homo sapiens GN=FAM208B PE=1 SV=1	0	6
Q9NU22	Midasin OS=Homo sapiens GN=MDN1 PE=1 SV=2	0	6
B4DLN1	Uncharacterized protein OS=Homo sapiens PE=2 SV=1	0	6
Q53H12	Acylglycerol kinase, mitochondrial OS=Homo sapiens GN=AGK PE=1 SV=2	0	6
O94964	Protein SOGA1 OS=Homo sapiens GN=SOGA1 PE=1 SV=2	0	6
Q8IWZ3	Ankyrin repeat and KH domain-containing protein 1 OS=Homo sapiens GN=ANKHD1 PE=1 SV=1	0	6
O60645	Exocyst complex component 3 OS=Homo sapiens GN=EXOC3 PE=1 SV=2	0	6
Q2NKX8	DNA excision repair protein ERCC-6-like OS=Homo sapiens GN=ERCC6L PE=1 SV=1	0	6
Q07864	DNA polymerase epsilon catalytic subunit A OS=Homo sapiens GN=POLE PE=1 SV=5	0	6
P07237	Protein disulfide-isomerase OS=Homo sapiens GN=P4HB PE=1 SV=3	0	6
P00367	Glutamate dehydrogenase 1, mitochondrial OS=Homo sapiens GN=GLUD1 PE=1 SV=2	0	6
G3V5T9	Cyclin-dependent kinase 2 OS=Homo sapiens GN=CDK2 PE=1 SV=1	0	6
Q15233	Non-POU domain-containing octamer-binding protein OS=Homo sapiens GN=NONO PE=1 SV=4	0	6
P12268	Inosine-5'-monophosphate dehydrogenase 2 OS=Homo sapiens GN=IMPDH2 PE=1 SV=2	0	6
P54578	Ubiquitin carboxyl-terminal hydrolase 14 OS=Homo sapiens GN=USP14 PE=1 SV=3	0	6
Q9H3U1	Protein unc-45 homolog A OS=Homo sapiens GN=UNC45A PE=1 SV=1	0	6
O75496	Geminin OS=Homo sapiens GN=GMNN PE=1 SV=1	0	6
Q9UQF2	C-Jun-amino-terminal kinase-interacting protein 1 OS=Homo sapiens GN=MAPK8IP1 PE=1 SV=1	0	6
Q9BTE3	Mini-chromosome maintenance complex-binding protein OS=Homo sapiens GN=MCMBP PE=1 SV=2	0	6

Q32Q12	Nucleoside diphosphate kinase OS=Homo sapiens GN=NME1-NME2 PE=1 SV=1	0	6
O60216	Double-strand-break repair protein rad21 homolog OS=Homo sapiens GN=RAD21 PE=1 SV=2	0	6
Q96EA4	Protein Spindly OS=Homo sapiens GN=SPDL1 PE=1 SV=2	0	6
P46977	Dolichyl-diphosphooligosaccharide--protein glycosyltransferase subunit STT3A OS=Homo sapiens GN=STT3A PE=1 SV=2	0	6
O75179	Ankyrin repeat domain-containing protein 17 OS=Homo sapiens GN=ANKRD17 PE=1 SV=3	0	6
Q9NQC8	Intraflagellar transport protein 46 homolog OS=Homo sapiens GN=IFT46 PE=1 SV=1	0	6
A0A024QZP7	Cyclin-dependent kinase 1 OS=Homo sapiens GN=CDK1 PE=1 SV=1	0	6
Q15397	Pumilio homolog 3 OS=Homo sapiens GN=PUM3 PE=1 SV=3	0	6
P36542	ATP synthase subunit gamma, mitochondrial OS=Homo sapiens GN=ATP5C1 PE=1 SV=1	0	6
Q9UEG4	Zinc finger protein 629 OS=Homo sapiens GN=ZNF629 PE=1 SV=2	0	6
Q9Y5A9	YTH domain-containing family protein 2 OS=Homo sapiens GN=YTHDF2 PE=1 SV=2	0	6
P61513	60S ribosomal protein L37a OS=Homo sapiens GN=RPL37A PE=1 SV=2	0	6
Q92804	TATA-binding protein-associated factor 2N OS=Homo sapiens GN=TAF15 PE=1 SV=1	0	6
Q9P003	Protein cornichon homolog 4 OS=Homo sapiens GN=CNIH4 PE=1 SV=1	0	6
Q9H000	Probable E3 ubiquitin-protein ligase makorin-2 OS=Homo sapiens GN=MKRN2 PE=1 SV=2	0	6
Q13257	Mitotic spindle assembly checkpoint protein MAD2A OS=Homo sapiens GN=MAD2L1 PE=1 SV=1	0	6
J3KQN4	60S ribosomal protein L36a OS=Homo sapiens GN=RPL36A PE=3 SV=1	0	6
A0A0J9YY99	Uncharacterized protein (Fragment) OS=Homo sapiens PE=1 SV=1	0	6
Q8WXE1	ATR-interacting protein OS=Homo sapiens GN=ATRIP PE=1 SV=1	0	6
P13804	Electron transfer flavoprotein subunit alpha, mitochondrial OS=Homo sapiens GN=ETFA PE=1 SV=1	0	5
E9PJ55	T-complex protein 11-like protein 1 OS=Homo sapiens GN=TCP11L1 PE=1 SV=1	0	5
D6RIT2	Heterogeneous nuclear ribonucleoprotein H (Fragment) OS=Homo sapiens GN=HNRNPH1 PE=1 SV=1	0	5
Q8TEX9	Importin-4 OS=Homo sapiens GN=IPO4 PE=1 SV=2	0	5
Q504Q3	PAB-dependent poly(A)-specific ribonuclease subunit PAN2 OS=Homo sapiens GN=PAN2 PE=1 SV=3	0	5
E9PD50	Protein SMG7 OS=Homo sapiens GN=SMG7 PE=1 SV=1	0	5
Q6Y7W6	PERQ amino acid-rich with GYF domain-containing protein 2 OS=Homo sapiens GN=GIGYF2 PE=1 SV=1	0	5
P32322	Pyrroline-5-carboxylate reductase 1, mitochondrial OS=Homo sapiens GN=PYCR1 PE=1 SV=2	0	5
P84085	ADP-ribosylation factor 5 OS=Homo sapiens GN=ARF5 PE=1 SV=2	0	5

P35222	Catenin beta-1 OS=Homo sapiens GN=CTNNB1 PE=1 SV=1	0	5
P42167	Lamina-associated polypeptide 2, isoforms beta/gamma OS=Homo sapiens GN=TMPO PE=1 SV=2	0	5
Q9Y5J1	U3 small nucleolar RNA-associated protein 18 homolog OS=Homo sapiens GN=UTP18 PE=1 SV=3	0	5
Q86XI2	Condensin-2 complex subunit G2 OS=Homo sapiens GN=NCAPG2 PE=1 SV=1	0	5
Q9Y4W6	AFG3-like protein 2 OS=Homo sapiens GN=AFG3L2 PE=1 SV=2	0	5
Q5T3I0	G patch domain-containing protein 4 OS=Homo sapiens GN=GPATCH4 PE=1 SV=2	0	5
P51116	Fragile X mental retardation syndrome-related protein 2 OS=Homo sapiens GN=FXR2 PE=1 SV=2	0	5
A0A0B4J2E5	Uncharacterized protein OS=Homo sapiens PE=1 SV=1	0	5
Q9NVE7	Pantothenate kinase 4 OS=Homo sapiens GN=PANK4 PE=1 SV=1	0	5
Q96GA3	Protein LTV1 homolog OS=Homo sapiens GN=LTV1 PE=1 SV=1	0	5
Q9BQ39	ATP-dependent RNA helicase DDX50 OS=Homo sapiens GN=DDX50 PE=1 SV=1	0	5
Q2NL82	Pre-rRNA-processing protein TSR1 homolog OS=Homo sapiens GN=TSR1 PE=1 SV=1	0	5
Q8TD19	Serine/threonine-protein kinase Nek9 OS=Homo sapiens GN=NEK9 PE=1 SV=2	0	5
Q9H9T3	Elongator complex protein 3 OS=Homo sapiens GN=ELP3 PE=1 SV=2	0	5
P35241	Radixin OS=Homo sapiens GN=RDX PE=1 SV=1	0	5
Q7Z5K2	Wings apart-like protein homolog OS=Homo sapiens GN=WAPL PE=1 SV=1	0	5
A0A087WVQ6	Clathrin heavy chain OS=Homo sapiens GN=CLTC PE=1 SV=1	0	5
Q7Z7A1	Centriolin OS=Homo sapiens GN=CNTRL PE=1 SV=2	0	5
O00743	Serine/threonine-protein phosphatase 6 catalytic subunit OS=Homo sapiens GN=PPP6C PE=1 SV=1	0	5
O43347	RNA-binding protein Musashi homolog 1 OS=Homo sapiens GN=MSI1 PE=1 SV=1	0	5
Q53GQ0	Very-long-chain 3-oxoacyl-CoA reductase OS=Homo sapiens GN=HSD17B12 PE=1 SV=2	0	5
Q12931	Heat shock protein 75 kDa, mitochondrial OS=Homo sapiens GN=TRAP1 PE=1 SV=3	0	5
O76031	ATP-dependent Clp protease ATP-binding subunit clpX-like, mitochondrial OS=Homo sapiens GN=CLPX PE=1 SV=2	0	5
Q9UG63	ATP-binding cassette sub-family F member 2 OS=Homo sapiens GN=ABCF2 PE=1 SV=2	0	5
P62140	Serine/threonine-protein phosphatase PP1-beta catalytic subunit OS=Homo sapiens GN=PPP1CB PE=1 SV=3	0	5
P78347	General transcription factor II-I OS=Homo sapiens GN=GTF2I PE=1 SV=2	0	5
O75152	Zinc finger CCCH domain-containing protein 11A OS=Homo sapiens GN=ZC3H11A PE=1 SV=3	0	5
P35606	Coatomer subunit beta' OS=Homo sapiens GN=COPB2 PE=1 SV=2	0	5

Q9H1A4	Anaphase-promoting complex subunit 1 OS=Homo sapiens GN=ANAPC1 PE=1 SV=1	0	5
Q9NRF8	CTP synthase 2 OS=Homo sapiens GN=CTPS2 PE=1 SV=1	0	5
Q96D53	AarF domain-containing protein kinase 4 OS=Homo sapiens GN=ADCK4 PE=1 SV=2	0	5
O95721	Synaptosomal-associated protein 29 OS=Homo sapiens GN=SNAP29 PE=1 SV=1	0	5
Q9Y2P8	RNA 3'-terminal phosphate cyclase-like protein OS=Homo sapiens GN=RCL1 PE=1 SV=3	0	5
P35232	Prohibitin OS=Homo sapiens GN=PHB PE=1 SV=1	0	5
O95613	Pericentrin OS=Homo sapiens GN=PCNT PE=1 SV=4	0	5
Q13155	Aminoacyl tRNA synthase complex-interacting multifunctional protein 2 OS=Homo sapiens GN=AIMP2 PE=1 SV=2	0	5
Q9Y619	Mitochondrial ornithine transporter 1 OS=Homo sapiens GN=SLC25A15 PE=1 SV=1	0	5
Q9NR09	Baculoviral IAP repeat-containing protein 6 OS=Homo sapiens GN=BIRC6 PE=1 SV=2	0	5
Q13838	Spliceosome RNA helicase DDX39B OS=Homo sapiens GN=DDX39B PE=1 SV=1	0	5
Q9Y4E8	Ubiquitin carboxyl-terminal hydrolase 15 OS=Homo sapiens GN=USP15 PE=1 SV=3	0	5
Q14152	Eukaryotic translation initiation factor 3 subunit A OS=Homo sapiens GN=EIF3A PE=1 SV=1	0	5
Q9BZF1	Oxysterol-binding protein-related protein 8 OS=Homo sapiens GN=OSBPL8 PE=1 SV=3	0	5
Q96BY7	Autophagy-related protein 2 homolog B OS=Homo sapiens GN=ATG2B PE=1 SV=5	0	5
P50750	Cyclin-dependent kinase 9 OS=Homo sapiens GN=CDK9 PE=1 SV=3	0	5
Q9HC21	Mitochondrial thiamine pyrophosphate carrier OS=Homo sapiens GN=SLC25A19 PE=1 SV=1	0	5
Q16513	Serine/threonine-protein kinase N2 OS=Homo sapiens GN=PKN2 PE=1 SV=1	0	5
P24666	Low molecular weight phosphotyrosine protein phosphatase OS=Homo sapiens GN=ACP1 PE=1 SV=3	0	5
Q9BQ04	RNA-binding protein 4B OS=Homo sapiens GN=RBM4B PE=1 SV=1	0	5
Q14145	Kelch-like ECH-associated protein 1 OS=Homo sapiens GN=KEAP1 PE=1 SV=2	0	5
O94927	HAUS augmin-like complex subunit 5 OS=Homo sapiens GN=HAUS5 PE=1 SV=2	0	5
Q63ZY3	KN motif and ankyrin repeat domain-containing protein 2 OS=Homo sapiens GN=KANK2 PE=1 SV=1	0	5
Q9Y4R8	Telomere length regulation protein TEL2 homolog OS=Homo sapiens GN=TELO2 PE=1 SV=2	0	5
Q99623	Prohibitin-2 OS=Homo sapiens GN=PHB2 PE=1 SV=2	0	5
Q9NWK9	Box C/D snoRNA protein 1 OS=Homo sapiens GN=ZNHIT6 PE=1 SV=1	0	5
Q9UNX4	WD repeat-containing protein 3 OS=Homo sapiens GN=WDR3 PE=1 SV=1	0	5
Q6P4A7	Sideroflexin-4 OS=Homo sapiens GN=SFXN4 PE=1 SV=1	0	5

O95163	Elongator complex protein 1 OS=Homo sapiens GN=IKBKAP PE=1 SV=3	0	5
Q9BWF3	RNA-binding protein 4 OS=Homo sapiens GN=RBM4 PE=1 SV=1	0	5
P62306	Small nuclear ribonucleoprotein F OS=Homo sapiens GN=SNRPF PE=1 SV=1	0	5
G3XAI2	Laminin subunit beta-1 OS=Homo sapiens GN=LAMB1 PE=1 SV=1	0	5
Q3L8U1	Chromodomain-helicase-DNA-binding protein 9 OS=Homo sapiens GN=CHD9 PE=1 SV=2	0	5
P00403	Cytochrome c oxidase subunit 2 OS=Homo sapiens GN=MT-CO2 PE=1 SV=1	0	5
Q8IWR0	Zinc finger CCCH domain-containing protein 7A OS=Homo sapiens GN=ZC3H7A PE=1 SV=1	0	5
Q92901	60S ribosomal protein L3-like OS=Homo sapiens GN=RPL3L PE=2 SV=3	0	5
Q8TAG9	Exocyst complex component 6 OS=Homo sapiens GN=EXOC6 PE=1 SV=3	0	5

Supplementary Table 2: List of primers used

Plasmid	Insert	Amino Acids	GenBank	Vector	Sequence (5'->3')
pDONR221-CEP164F	CEP164F	1-1460 aa	NM_014956	pDONR221	GGGGACAAGTTTGTACAAAAAAGCAGGCTTCatggctggacgacccctcc
					GGGGACCACTTTGTACAAGAAAGCTGGGTCtcagaagcgatacaccttc
pDONR221-CEP164N	CEP164N	1-580 aa	NM_014956	pDONR221	GGGGACAAGTTTGTACAAAAAAGCAGGCTTCatggctggacgacccctcc
					GGGGACCACTTTGTACAAGAAAGCTGGGTCTCAaggctctgtggatcgcacc
pDONR221-CEP164M	CEP164M	581-1045 aa	NM_014956	pDONR221	GGGGACAAGTTTGTACAAAAAAGCAGGCTTCgtggctcccccagagcagc
					GGGGACCACTTTGTACAAGAAAGCTGGGTCTCAttctctcaacaggtgctgc
pDONR221-CEP164C	CEP164C	1046-1460 aa	NM_014956	pDONR221	GGGGACAAGTTTGTACAAAAAAGCAGGCTTCgtgacagttgaggaaaata
					GGGGACCACTTTGTACAAGAAAGCTGGGTCtcagaagcgatacaccttc
pDONR221-CEP164N1	CEP164N1	1-298 aa	NM_014956	pDONR221	GGGGACAAGTTTGTACAAAAAAGCAGGCTTCatggctggacgacccctcc
					GGGGACCACTTTGTACAAGAAAGCTGGGTCttagccaacagcactgctcagactgct
pDONR221-CEP164N2	CEP164N2	299-580 aa	NM_014956	pDONR221	GGGGACAAGTTTGTACAAAAAAGCAGGCTTCaaagggcgacagggaagtggagca
					GGGGACCACTTTGTACAAGAAAGCTGGGTCTCAaggctctgtggatcgcacc
pDONR221-CCDC92F	CCDC92F	1-314 aa	NM_001418046	pDONR221	GGGGACAAGTTTGTACAAAAAAGCAGGCTTCatggcagccacaaacctggagaacc
					GGGGACCACTTTGTACAAGAAAGCTGGGTCTTAtcacacagttcggtctgtccctgag
pDONR221-CCDC92N	CCDC92N	1-160 aa	NM_001418046	pDONR221	GGGGACAAGTTTGTACAAAAAAGCAGGCTTCatggcagccacaaacctggagaacc
					GGGGACCACTTTGTACAAGAAAGCTGGGTCTTAtcaatccgaggtgccacttgagct
pDONR221-CCDC92C	CCDC92C	161-314 aa	NM_001418046	pDONR221	GGGGACAAGTTTGTACAAAAAAGCAGGCTTCgccagccctgccggcagtc
					GGGGACCACTTTGTACAAGAAAGCTGGGTCTTAtcacacagttcggtctgtcc
pDONR221-CCDC92C1	CCDC92C1	161-237 aa	NM_001418046	pDONR221	GGGGACAAGTTTGTACAAAAAAGCAGGCTTCgccagccctgccggcagtccc
					GGGGACCACTTTGTACAAGAAAGCTGGGTCCTActcagcagactcccgggccag
pDONR221-CCDC92C2	CCDC92C2	238-314 aa	NM_001418046	pDONR221	GGGGACAAGTTTGTACAAAAAAGCAGGCTTCgtccagctcaaggagcggcct
					GGGGACCACTTTGTACAAGAAAGCTGGGTCCTAcacagttcggtctgtccctga
pDONR221-CCDC92ΔHCR1	CCDC92ΔHCR1	1-181&195-314 aa	NM_001418046	pDONR221	CCCAAGGACAAGCTGgccccactgcaccccgagtttgaggag
					ggggtgcagtggggcCAGCTTGTCCTTGGGGGGTGTGGGTTT
pDONR221-CCDC92ΔHCR2	CCDC92ΔHCR2	1-219&234-314 aa	NM_001418046	pDONR221	CTCCTTCGGGAGCCTgagtctgctgaggtccagctcaaggag
					gacctcagcagactcAGGCTCCCGAAGGAGGAGTTTGCGGCT
pDONR221-IFT74F	IFT74F	1-600 aa	NM_026319	pDONR221	GGGGACAAGTTTGTACAAAAAAGCAGGCTTCatggccagtaatcacaaat
					GGGGACCACTTTGTACAAGAAAGCTGGGTCTTAtcagcttctgctggcattatgt

pDONR221-IFT74N	IFT74N	1-280 aa	NM_026319	pDONR221	GGGGACAAGTTTGTACAAAAAAGCAGGCTTCatggccagtaatcacaaat<br>GGGGACCACTTTGTACAAGAAAGCTGGGTCTTAtcatttttcatacagcaatacag
pDONR221-IFT74M	IFT74M	281-400 aa	NM_026319	pDONR221	GGGGACAAGTTTGTACAAAAAAGCAGGCTTCctttatgagttagagtccca<br>GGGGACCACTTTGTACAAGAAAGCTGGGTCTTAtcagcaatgctctaacagtgtga
pDONR221-IFT74C	IFT74C	401-600 aa	NM_026319	pDONR221	GGGGACAAGTTTGTACAAAAAAGCAGGCTTCagtcggaatataaatcgtat<br>GGGGACCACTTTGTACAAGAAAGCTGGGTCTTAtcagcttctgctggcattatgt
pDONR221-Luci	Luciferase	1-550 aa	MK484107	pDONR221	GGGGACAAGTTTGTACAAAAAAGCAGGCTTCatggaagacgccaaaaaca<br>GGGGACCACTTTGTACAAGAAAGCTGGGTCTTAttacacggcgatctttccg
pGEX-4T-1-CEP164N1	CEP164N1	1-298 aa	NM_014956	pGEX-4T-1	GATCTGGTTCCGCGTGGATCCatggctggacgacccctccg<br>CTCGAGTCGACCCGGGAATTCTCAgccaacagcactgctcagac
pGEX-4T-1-CEP164M	CEP164M	581-1045 aa	NM_014956	pGEX-4T-1	GATCTGGTTCCGCGTGGATCCgtggctcccccagagcagctc<br>GAGTCGACCCGGGAATTCtcattctctcaacaggtgctgctt
pGEX-4T-1-CEP164C	CEP164C	1046-1460 aa	NM_014956	pGEX-4T-1	GATCTGGTTCCGCGTGGATCCgtgacagttgaggaaaataat<br>GAGTCGACCCGGGAATTCtcagaagcgatacaccttcactct
pGEX-4T-1-IFT74M	IFT74M	281-400 aa	NM_026319	pGEX-4T-1	GATCTGGTTCCGCGTGGATCCctttatgagttagagtccca<br>CTCGAGTCGACCCGGGAATTCTCAgcaatgctctaacagtgtga
pGEX-4T-1-IFT74C	IFT74C	401-600 aa	NM_026319	pGEX-4T-1	GATCTGGTTCCGCGTGGATCCagtcggaatataaatcgtat<br>CTCGAGTCGACCCGGGAATTCtcagcttctgctggcattatg
pET-32a-CCDC92N	CCDC92N	1-160 aa	NM_001418046	pET-32a	GCCATGGCTGATATCGGATCCatggcagccacaaacctggag<br>CTCGAGTGCGGCCGCAAGCTTatccgaggtgccacttgagct
pET-32a-CCDC92C1	CCDC92C1	161-237 aa	NM_001418046	pET-32a	ATGGCTGATATCGGATCCatggccagccctgccggcagtccc<br>CTCGAGTGCGGCCGCAAGCTTctcagcagactcccgggccag
PET-28a-CFAP77	CFAP77	106-260aa	NM_001166705	PET-28a	TGGACAGCAAATGGGTCGCGGATCCtttaagcagcaacctacc<br>TGGTGGTGGTGGTGGTGCTCGAGgaagccacatgatgtcag
PET-28a-CFAP276	CFAP276	1-168aa	NM_029314	PET-28a	TGGACAGCAAATGGGTCGCGGATCCatgcttcagcctcaggaaactttc<br>TGGTGGTGGTGGTGGTGCTCGAGggtggagaagaagccaccatcattt
PET-28a-SPMIP6	SPMIP6	52-246aa	NM_001048005	PET-28a	TGGACAGCAAATGGGTCGCGGATCCcaccgccacgtggaccctgaggcc<br>TGGTGGTGGTGGTGGTGCTCGAGttgcattcctggccggtgaccgaa

	Primers	Sequence (5'->3')
Genotyping qPCR	Ccdc92KO-F1	GTAGGTGGAATGCCAAGACAGG
	Ccdc92KO-R1	CAAAAAGACTCGGAGGAAAAGG
	Ccdc92KO-F2	TAGTGACACAATGCTACAATGGTG
	Ccdc92KO-R2	CGAGGCTGCTGCTCTGCTC
	Ccdc92-F	ACTGCACAGATCTAACATATGAGC
	Ccdc92-R	CAGTTTGTGGCTCTTCACCT
	Gapdh-F	AGGTCGGTGTGAACGGATTTG
	Gapdh-R	TGTAGACCATGTAGTTGAGGTCA

Supplementary Table 3: List of antibodies used

Primary antibodies					
**Antigen**	**Isotype**	**Supplier**	**Cat.#**	**WB**	**IF**
Rat anti GFP antibody	IgG2a,k	BioLegend	338002		1:200
Rabbit anti IFT57 antibody	IgG	Proteintech	11083-1-AP		1:200
Rabbit anti IFT20 antibody	IgG	Proteintech	13615-1-AP		1:200
Rabbit anti IFT140 antibody	IgG	Proteintech	17460-1-AP		1:200
Rabbit anti IFT88 antibody	IgG	Proteintech	13967-1-AP		1:200
Rabbit anti IFT80 antibody	IgG	Proteintech	25230-1-AP		1:200
Rabbit anti IFT74 antibody	IgG	Proteintech	27334-2-AP	1:1000	1:200
Rabbit anti IFT81 antibody	IgG	Proteintech	11744-1-AP	1:1000	1:200
Rabbit anti ENKUR antibody	IgG	Proteintech	26440-1-AP	1:1000	
Rabbit anti TEKT1 antibody	IgG	Proteintech	18968-1-AP	1:1000	
Rabbit anti TEKT3 antibody	IgG	Proteintech	12959-1-AP	1:1000	
Rabbit anti CEP164 antibody	IgG	Proteintech	22227-1-AP	1:1000	
Rabbit anti CCDC181 antibody	IgG	Thermo Fisher	PA555579	1:2000	
Rabbit anti CCDC92 antibody	IgG	home-made		1:1000	1:200
Rat anti CFAP77 antibody	IgG	home-made		1:1000	
Rat anti CFAP276 antibody	IgG	home-made			1:200
Rat anti SPMIP6 antibody	IgG	home-made			1:200
Chicken anti CEP164 antibody	IgY	home-made			1:1000
Mouse anti α-Tubulin antibody	IgG1	Sigma	T6199		1:1000
Mouse anti acetylated tubulin antibody	IgG2b	Sigma	T6793		1:1000
Rabbit anti FLAG antibody	IgG	Sigma	F7425	1:2000	
HRP-Conjugated GFP Tag Antibody	IgG2a	Proteintech	HRP-66002	1:5000	
HRP-Conjugated His-Tag Antibody	IgG1	Proteintech	HRP-66005	1:5000	

Secondary antibodies					
Name	Label or Dye	Supplier	Cat.#	WB	IF
Goat anti-Mouse IgG (H+L)	HRP	Invitrogen	31430	1:20000	
Goat anti-Rat IgG (H+L)	HRP	Invitrogen	31470	1:5000	
Goat anti-Rabbit IgG (H+L)	HRP	Invitrogen	31460	1:20000	
Donkey anti-Rat IgG（H+L)	Alexa Fluor 488	Thermo Fisher	A48269		1:1000
Goat anti-Rabbit IgG（H+L)	Alexa Fluor 555	Thermo Fisher	A-21428		1:1000
Donkey anti-Mouse IgG（H+L)	Dylinght 405	Jackson ImmunoResearch	715-475-151		1:200
Donkey anti-Chicken IgY (H+L)	Alexa Fluor 647	Jackson ImmunoResearch	703-605-155		1:1000

Supplementary methods

Cell culture, transfection, and lentiviral infection

HEK293T (CRL-11268, ATCC) and IMCD3 (CRL-2123, ATCC) cells were cultured in Dulbecco's modified Eagle's medium (DMEM; C11995500BT, Thermo Fisher) supplemented with 10% fetal bovine serum (FBS; 04-001-1ACS, Biological Industries), 1% penicillin/streptomycin (P1400, Solarbio) and 2 mM L-alanyl-l-glutamine (G0190, Solarbio). hTERT-RPE1 cells (CRL-400, ATCC) were grown in Dulbecco's Modified Eagle Medium/Nutrient Mixture F-12 (DMEM/F-12; C11330500BT, Thermo Fisher) supplemented with 10% FBS, 1% penicillin/streptomycin, and 2 mM L-alanyl-l-glutamine. Additionally, 10 μg/ml hygromycin B (60224ES03, Yeasen Biotech) was added to the hTERT-RPE1 culture medium. All cell lines were routinely tested for mycoplasmas.

HEK293T cells at about 70% confluency were transfected with indicated plasmids and 1 mg/ml polyethylenimine (PEI; 23966, Polysciences) at a ratio of 1:1.5, and cell samples were harvested 48 hours after transfection and subjected to the co-immunoprecipitation (Co-IP) assay. Lentiviral particles were produced as described previously. Briefly, HEK 293T cells were transfected with a lentiviral plasmid, pCMV-D8.9, and pCMV-VSVG at the ratio 5:3:2 using PEI for 48 hours. The culture medium containing lentiviral particles was collected and added to the hTERT-RPE1 culture medium at a 1:20 dilution. hTERT-RPE1 and IMCD3 cells were serum-starved for 24 hours to induce cilia formation.

Immunoprecipitations

Coimmunoprecipitation was conducted as described previously. In brief, HEK293T cells were transiently transfected, cultured for 48 hours, washed with PBS, and lysed in high-salt lysis buffer (1% NP-40, 500 mM NaCl, 50 mM Hepes [PH 7.8], 5 mM EDTA) containing 50 mM NaF, 1 mM Na3VO4, 3 mM DTT, 1 mM PMSF and complete protease inhibitors (539134, Calbiochem). Lysates were further cleared by centrifugation at 14000 g for 10 minutes, and supernatants were incubated with GFP-Nanoab-Agarose beads (GNA-50-1000, Lablead) for 4 hours at 4°C with gentle rotation. Beads were washed in lysis buffer, eluted in sample buffer, and analyzed by sodium dodecyl-sulfate polyacrylamide gel electrophoresis (SDS-PAGE).

Live cell imaging

Live imaging was conducted on a spinning disk confocal microscope with a Zeiss Plan-Apochromat 63 x / 1.4 or 100 x / 1.46 oil immersion objective (Intelligent Imaging Innovations). hTERT-RPE1 cells expressing GFP-CCDC92 were cultured in a glass-bottom dish. For imaging the movement of GFP-CCDC92 bulges, image acquisition was recorded every 3 minutes using a HAMAMATSU digital CMOS camera (C11440, HAMAMATSU Photonics). Images were processed using ImageJ (National Institutes of Health) or the SlideBook software (Intelligent Imaging Innovations).

GST pull-down assay

His-CCDC92N and His-CCDC92C1 proteins were purified using Ni-NTA agarose beads (30210, Qiagen). The GST-tagged CEP164 and IFT74 truncated proteins were expressed using the BL21-Codon Plus (DE3)-RIPL strain. The glutathione sepharose beads (GE17075601, Sigma) were incubated with bacterial lysate at 4°C for 4 hours and washed three times with high-salt lysis buffer. 1 ml lysis buffer containing each purified His tagged protein was incubated with beads for 4 hours at 4°C. Beads were washed three times with lysis buffer, eluted in sample buffer, and analyzed by SDS-PAGE.

Animals

For fertility testing, four WT and four *Ccdc92* KO mice (ten weeks of age) were each paired continuously with one ten-week-old WT mouse for three months. During the test, the timing and size of the litters were recorded, and the pups were removed to leave each mating pair to continue mating until the end of the test. The *in vitro* fertilization (IVF) and the intracytoplasmic sperm injection (ICSI) were performed as previously reported. GemPharmatech carried out IVF and ICSI experiments, and we analyzed the results.

For the Astral DIA analysis, individual testicle isolated from ten-week-old WT or *Ccdc92* KO mice was quick-frozen in liquid nitrogen and sampled separately. Proteins were extracted, digested, and analyzed by an asymmetric track lossless (Astral) mass analyzer (MajorBio, China). The principal component analysis (PCA) was performed using the prcomp function in R (version 4.1.1), the proteomic differential expression analysis of samples with biological replicates was carried out using EdgeR, and the functional enrichment analysis was conducted using Metascape. The proteomic analysis was visualized using the ggplot2 and ggrepel packages in R.

Quantitative real-time PCR

Tissues were isolated from WT mice and snap-frozen in liquid nitrogen. RNA was extracted from each tissue using the TransZol Up Plus RNA kit (ER501-01-V2, TransGen Biotech) and transcribed to cDNA using the one-step RT-gDNA digestion Super Mix (11141ES60, Yeasen Biotech). The quantitative real-time PCR was performed using the SYBR Green PCR Kit (11185ES08, Yeasen Biotech). *Gapdh* was utilized for normalization. The primers used are listed in Supplementary Table S2.

Histological analysis

Eight-week-old mice were transcardially perfused with 50 ml PBS and 50 ml 4% paraformaldehyde (PFA) in PBS. Epididymis and testis were dissected and immediately postfixed in 4% PFA and Bouin's fixative (HT10132, Sigma) for 24 hours, respectively. Samples were dehydrated with an automatic tissue processor (HistoCore PEARL, Leica), embedded in paraffin, and sectioned at a 5-micrometer thickness on a rotary microtome. Sample sections were laid on the surface of the slide, cleaned with xylene, rehydrated, and subjected to staining.

For the hematoxylin and eosin (HE) staining, sections were treated with a hematoxylin staining solution (E607317, Sangon Biotech) for 30 seconds, rinsed with tap water for 3 minutes, and then placed in 1% hydrochloric acid ethanol for 5 seconds, followed by rinsing with tap water until the blue color returned. The sections were then put in a 0.25% Eosin Y alcoholic solution for 30 seconds and immediately rinsed with 90% ethanol to remove excess staining. After sequential dehydration with 90% ethanol, 100% ethanol (I), and 100% ethanol (II) for 5 minutes, the sections were placed in xylene for 10 minutes twice for transparency and mounted with the neutral resin (G8590, Solarbio). The slides were thoroughly air-dried in a fume hood.

For the periodic acid-Schiff (PAS) staining, the dewaxed sections were stained with the glycogen PAS staining kit (KGE1103-400, Nanjing KeyGen Biotech). Sections were rinsed with tap water for 5 minutes and then immersed in the hematoxylin staining solution for 30 seconds, rinsed with tap water for 3 minutes, immersed in 1% hydrochloric acid ethanol solution for 5 seconds, and then quickly rinsed in tap water for 15 minutes to restore the blue color. The subsequent gradient ethanol dehydration, xylene transparency, and sealing steps were the same as described in HE staining.

For the Giemsa staining of spermatozoa, cauda epididymal spermatozoa isolated from WT and *Ccdc92* KO mice were smeared on a slide, air-dried, and fixed with 4% PFA in PBS for 20

minutes. Samples were stained with the Giemsa staining solution (C0133, Beyotime Biotech) for 45 minutes, rinsed thoroughly with tap water, and air-dried in a fume hood before image acquisition.

Histologically stained sections were imaged using a 3D Histech digital slide scanner (Pannoramic MIDI, 3DHISTECH).

Scanning electron microscopy

Sperm was released from the cauda epididymis in PBS at 37°C for 30 min, and 200 μl of cell suspension was placed on poly-lysine-coated slides for 15 minutes and fixed with the fixative containing 2.5% glutaraldehyde (GA) and 4% PFA overnight. Samples were washed with 0.1 M phosphate buffer three times for 10 minutes each, post-fixed in 1% OsO_4 for 1 hour, and then dehydrated with a gradient of ethanol (50%, 70%, 80%, 90%, 100%, 100%, and 100%) for 10 minutes each. The samples were dried by critical point drying, gold-coated by the sputtering technique, and examined with a scanning electron microscope (TM3030, Hitachi Asia Ltd) at an accelerating voltage of 15 kV.

Transmission electron microscopy

Indicated mice were transcardially perfused with 50 ml PBS and 50 ml 4% PFA in PBS. Testis and epididymis were dissected and further fixed in 2.5% GA and 4% PFA for 2 hours at room temperature, followed by fixation at 4°C overnight. Samples were washed three times with PBS for 15 minutes each, followed by the treatment with 1% OsO4 for 1 hour at 4°C. The sample was dehydrated by graded acetone series (50%, 60%, 70%, 80%, 90%, 95%, 100%), embedded in Epon 812 resin, and polymerized. 60 nm ultrathin sections were stained with 2% uranyl acetate for 10 min and 1% lead citrate for 5 min. Images were obtained and analyzed using the HT-7800 transmission electron microscope (Hitachi Asia Ltd).

Supplementary video legends

Video S1 Sperm motilities in representative regions of WT and *Ccdc92* KO spermatozoa.
Mature spermatozoa released from the cauda epididymides of eight-week-old WT and *Ccdc92* KO mice were live imaged with the computer-assisted sperm analysis (CASA) image system. Image sequences are played back at 10 fps.

Video S2 GFP-CCDC92 displays a bidirectional movement along the ciliary axoneme.
hTERT-RPE1 cells co-expressing GFP-CCDC92 and SMO^{M2}-tRFP were serum-starved for 24 hours and live imaged with a spinning disk confocal microscope overnight. Selected image sequences are played back at 7 fps.

Video S3 GFP-CCDC92 can be released from the ciliary tip.
hTERT-RPE1 cells co-expressing GFP-CCDC92 and SMO^{M2}-tRFP were serum-starved for 24 hours and live imaged with a spinning disk confocal microscope overnight. Selected image sequences are played back at 3 fps.
